# Impact of hospital process reengineering on door-to-needle time for intravenous thrombolysis in acute ischemic stroke (PROMISE-CHINA): a multicenter prospective pre-post quasi-experimental study

**DOI:** 10.3389/fneur.2026.1746553

**Published:** 2026-04-10

**Authors:** Dawei Dong, Longyan Lu, Niu He, Junrun Zhang, Ziran Wang, Haiqin Xia, Xuwen Sun, Yu Geng, Hui Liang, Xiaoyuan Niu, Meng Zhang, Dongjuan Xu, Ning Li, Jianhua Li, Wanhua Wang, Min Zhang, Zhong Zhang, Yongjun Cao, Li Sun, Junshan Zhou, Weirong Li, Peilan Li, Jianhui Fu, Wei Qiu, Yanzhong Xue, Yumin Liu, Jinli Zhang, Weiwen Qiu, Yan Xing, Zhengqi Lu, Fuling Liu, Shixia Wang, Jilai Li, Dawei Zang, Junyan Liu, Juan Feng, Zhengshe Bao, Suping Zhang, Yongjun Wang, Xiufeng Xin, Huili Zhu, Anding Xu

**Affiliations:** 1 Department of Neurology and Stroke Center, the First Affiliated Hospital, Jinan University, Guangzhou, China; 2 Clinical Neuroscience Institute, Jinan University, Guangzhou, China; 3 Emergency Department, Linyi People's Hospital, Linyi, China; 4 Department of Neurology, The Six Hospital of Shanxi Medical University, Taiyuan, China; 5 Department of Neurology, Yantai Yuhuangding Hospital, Yantai, China; 6 Department of Neurology, Zhejiang Provincial People's Hospital, Affiliated People's Hospital, Hangzhou Medical College, Hangzhou, China; 7 Department of Neurology, Yantaishan Hospital, Yantai, China; 8 Department of Neurology, The First Hospital of Shanxi Medical University, Taiyuan, China; 9 Department of Neurology, Daping Hospital & Research Institute of Surgery, The Army Military Medical University, Chongqing, China; 10 Department of Neurology, Dongyang People's Hospital, Wenzhou Medical University, Jinhua, China; 11 Department of Neurology, Tieying Hospital of Fengtai District, Beijing, China; 12 Department of Neurology, First Hospital of Fangshan District, Beijing, China; 13 Department of Neurology, The First People's Hospital of Kunshan, Suzhou, China; 14 Department of Neurology, Qingdao Municipal Hospital, Qingdao, China; 15 Department of Neurology, The Third People's Hospital of Chengdu, Chengdu, China; 16 Department of Neurology, The Second Affiliated Hospital of Soochow University, Suzhou, China; 17 Department of Neurology, Qingdao Central Hospital, Qingdao University, Qingdao, China; 18 Department of Neurology, Nanjing First Hospital, Nanjing Medical University, Nanjing, China; 19 Department of Neurology, Taiyuan Central Hospital of Shanxi Medical University, Taiyuan, China; 20 Emergency Department, Beijing Boai Hospital & China Rehabilitation Research Center, Beijing, China; 21 Department of Neurology, Shanghai Pudong Hospital, Fudan University Pudong Medical Center, Shanghai, China; 22 Department of Neurology, The Third Affiliated Hospital of Sun Yat-sen University Lingnan Hospital, Guangzhou, China; 23 Department of Neurology, Linyi Central Hospital, Linyi, China; 24 Department of Neurology, Zhongnan Hospital of Wuhan University, Wuhan, China; 25 Department of Neurology, The People's Liberation Army No. 263 Hospital, Beijing, China; 26 Department of Neurology, Lishui Central Hospital, Lishui, China; 27 Department of Neurology, Aviation General Hospital of China Medical University & Beijing Institute of Translational Medicine, Chinese Academy of Sciences, Beijing, China; 28 Department of Neurology, The Third Affiliated Hospital of Sun Yat-sen University, Guangzhou, China; 29 Department of Neurology, Pingdu People's Hospital, Qingdao, China; 30 Department of Neurology, Neimenggu Baogang Hospital, Inner Mongolia Medical University, Baotou, China; 31 Department of Neurology, Aerospace Central Hospital, Beijing, China; 32 Department of Neurology, Tianjin First Central Hospital of Tianjin Medical University, Tianjin, China; 33 Department of Neurology, Third Hospital of Hebei Medical University, Shijiazhuang, China; 34 Department of Neurology, Shengjing Hospital of China Medical University, Shenyang, China; 35 Department of Neurology, Beijing Yanhua Hospital, Beijing, China; 36 Department of Neurology, Guangzhou Red Cross Hospital, Medical College, Jinan University, Guangzhou, China; 37 Beijing Tiantan Hospital, Capital Medical University, Beijing, China

**Keywords:** acute ischemic stroke, door-to-needle time, healthcare quality, hospital processes reengineering, rate of thrombolysis

## Abstract

**Background:**

Intravenous thrombolytic therapy significantly improves the prognosis of patients with acute ischemic stroke in a time-dependent manner. This study aims to evaluate the effectiveness of hospital process reengineering in reducing delays to intravenous thrombolysis in patients with acute ischemic stroke.

**Methods:**

This multicenter, prospective, nonrandomized quasi-experimental (pre-post) study included patients with acute ischemic stroke presenting within 3.5 h of symptom onset. Hospital process reengineering involved key measures such as pre-notification by emergency medical services, simultaneous activation of a multidisciplinary team, standardized communication, and regular feedback to streamline workflows. Data from pre-intervention (July 1–September 30, 2014, Q1) were compared to post-intervention (October 1, 2014–June 30, 2015, Q2–Q4). The primary outcomes included the door-to-needle time and its changes, the proportion of patients receiving intravenous thrombolysis, and the percentage of patients achieving a door-to-needle time <60 min.

**Results:**

A total of 2,059 acute ischemic stroke patients were included, with 535 in the pre-intervention period and 1,524 in the post-intervention period. Following the intervention, the median door-to-needle time significantly decreased from 73 to 63 min (*p* = 0.001); however, the thrombolysis rate remained statistically unchanged, with rates of 63.0% pre-intervention and 63.5% post-intervention (*p* = 0.849). Moreover, when the post-intervention period was subdivided into three quarters (Q2–Q4), there was a consistent downward trend in the median door-to-needle time (P for trend = 0.001). In addition, the percentage of patients achieving a door-to-needle time of less than 60 min increased from 31.5 to 40.5% (*p* = 0.003).

**Conclusion:**

Hospital process reengineering significantly improved door-to-needle time, highlighting the importance of optimizing workflows in acute stroke care. Although this study was conducted a decade ago, its findings continue to offer valuable insights and practical implications for underdeveloped regions and countries.

**Clinical trial registration:**

Clinicaltrials.gov, NCT02631317 (https://clinicaltrials.gov/study/NCT02631317).

## 1 Introduction

Guidelines recommend intravenous infusion of tissue plasminogen activator (IV-tPA) for patients with ischemic stroke within 4.5 h (1–3), targeting a door-to-needle time (DNT) of less than 60 min (4–6). However, the proportion of eligible patients treated with IV-tPA and those achieving a DNT < 60 min have been consistently and significantly lower in China compared to developed countries (7, 8). The Chinese National Stroke Registry (CNSR, 2012–2013) reported that 18% of patients with acute ischemic stroke (AIS), who presented within 2 h of symptom onset, received IV-tPA within 3 h, a stark contrast to the 60% reported in the “Get with the Guidelines” (GWTG) program in the United States (9, 10). Moreover, the median DNT in China was 115 min, with fewer than 10% of patients achieving a DNT < 60 min, compared to a median DNT of 78 min and 26.6% meeting the threshold in the United States (5, 8, 11). In 2011, the American Heart Association and American Stroke Association introduced ten evidence-based strategies—including emergency medical service (EMS) prenotification, single-call stroke team activation, rapid brain imaging, standardized protocols, premixed tPA, a team-based approach, and prompt feedback—to reduce DNT in AIS patients (5). However, due to unique systemic challenges in China, many of these measures are not directly applicable; the lower thrombolysis rates may be attributable to more complex consent procedures, patient or family refusal, diagnostic delays, lack of real-time feedback, insufficient urgency awareness among healthcare professionals, and limited infrastructure. To address these issues, we initiated the PROMISE-CHINA study in 2014–2015, which based on business process reengineering theory and established practices (5, 12), implemented hospital reengineering processes (HRP) for IV-tPA. Unlike standard care, which typically follows conventional protocols without systematic optimization, HRP involves targeted process reengineering strategies designed to overcome the specific barriers present in the Chinese healthcare system. This study aimed to assess the impact of HPR on workflow efficiency in acute ischemic stroke, with a primary focus on reducing DNT, and to examine its effects on intravenous thrombolysis utilization and timely treatment delivery.

## 2 Materials and methods

### 2.1 Study design

Between July 1, 2014, and June 30, 2015, we conducted a multicenter, prospective, nonrandomized quasi-experimental (pre-post) study to evaluate the impact of HPR on patients with AIS who presented within 3.5 h of symptom onset. To account for 1 h allocated to in-hospital preparation, we deliberately enrolled only those AIS patients admitted within 3.5 h instead of the conventional 4.5-h window. The study included both patients who received intravenous thrombolysis treatment and those who did not. Participating centers had to meet the following criteria: (1) voluntary participation with consistent and complete reporting of eligible cases; (2) possess the necessary qualifications and experience for intravenous rt-PA thrombolysis; and (3) successfully revamp their thrombolysis process according to the established framework for process reengineering developed by the research committee. Data from sub-centers during the pre-intervention period (July 1, 2014, to September 30, 2014) served as the control group, while from October 1, 2014, to June 30, 2015, the HPR strategy was implemented. All centers used a uniform electronic database to record demographic and clinical information, including diagnosis, treatments, and key time points (e.g., stroke symptom onset, arrival time, imaging, and intravenous thrombolysis). Patients were further grouped by quarter: Q1 for the pre-intervention period and Q2–Q4 for the post-intervention period. The study was registered in July 2014 (https://register.clinicaltrials.gov, ID: NCT02631317).

### 2.2 Study participants

**Figure 1:**
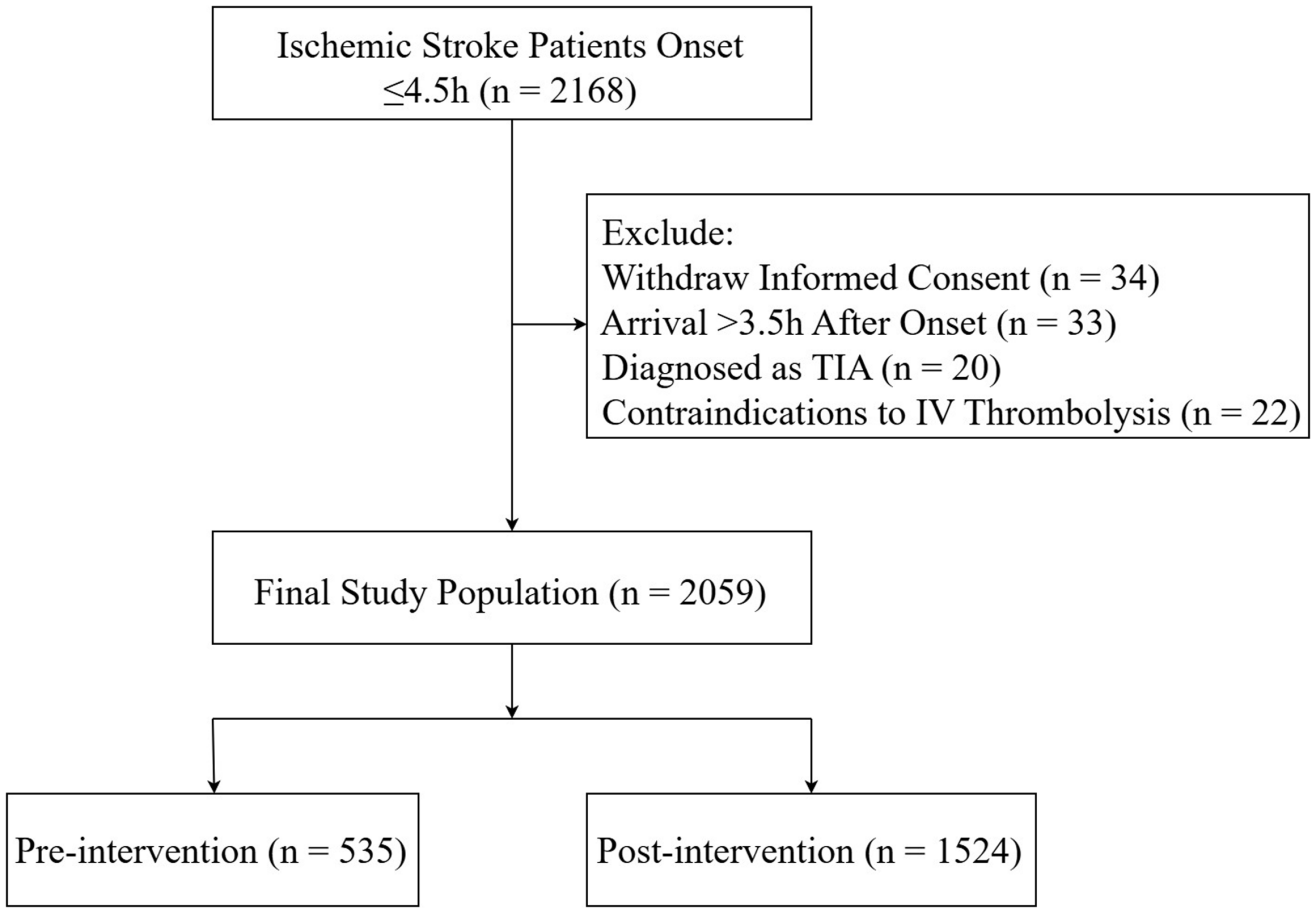
Flowchart.

Inclusion criteria were (1) patients with AIS within 3.5 h of symptom onset; and (2) written informed consent obtained from the patient or a legally authorized representative. Exclusion criteria included: (1) patients with complete resolution of neurological deficits, consistent with a transient ischemic attack (TIA); (2) patients with radiologically confirmed silent cerebral infarctions without clinical manifestations; and (3) patients with contraindications to intravenous thrombolytic therapy. This study was approved by the Institutional Review Board of the First Affiliated Hospital of Jinan University (ethical approval number: 2014-LSPY-026). Detailed information is presented in Figure 1.

### 2.3 Intervention

**Figure 2:**
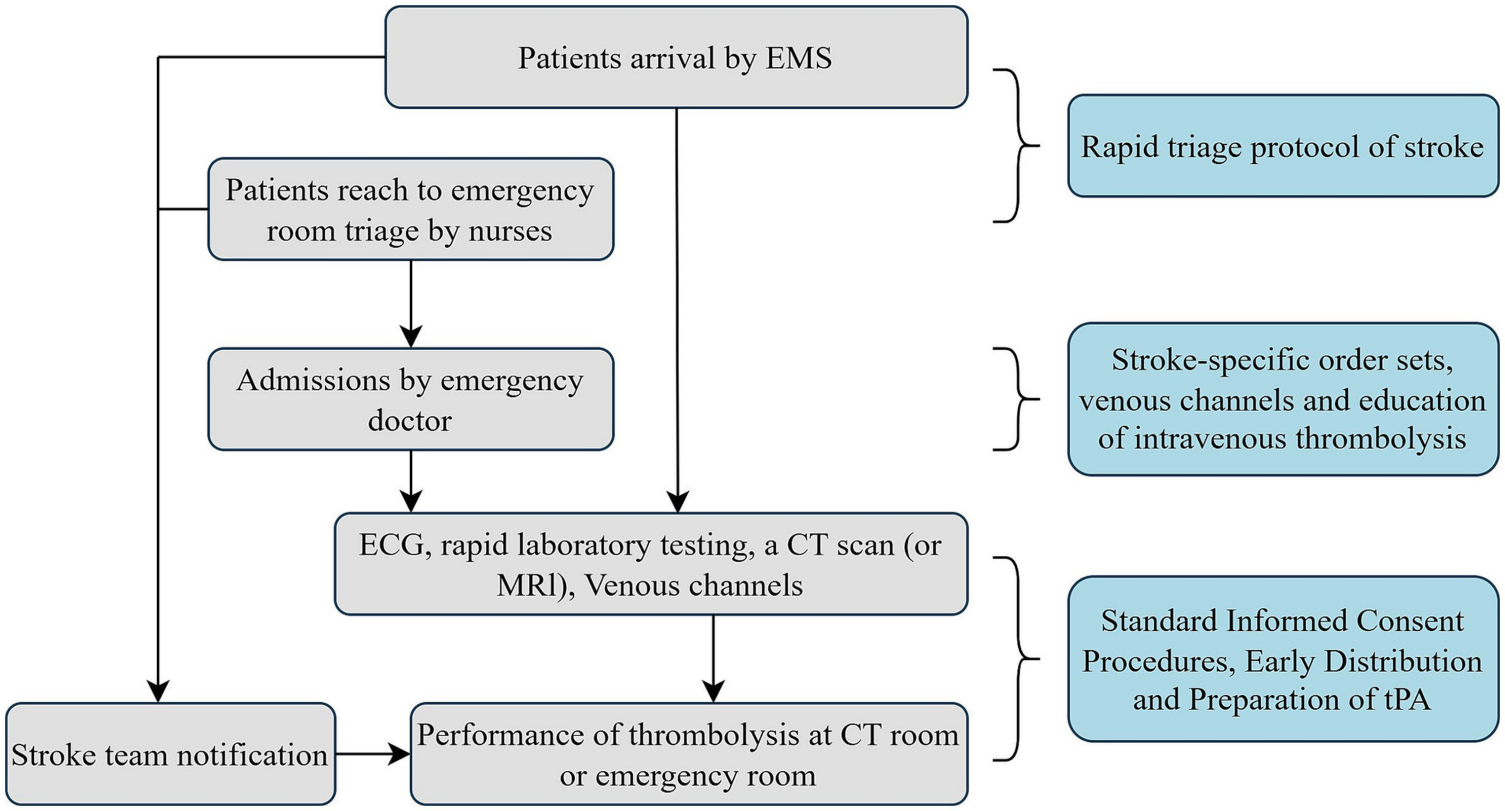
Practice strategies of hospital processes reengineering for intravenous thrombolysis. EMS, emergency medical service; ECG, electrocardiograph.

Based on the Business Process Reengineering (BPR) theory, which focuses on the radical redesign of workflows to improve efficiency, and published practice strategies (5, 12), the study committee developed a set of HPR for AIS patients eligible for intravenous thrombolysis treatment (Figure 2). A more detailed description of BPR is provided in Section 1.3 of the study Protocol. The key measures implemented included: (1) establishing a dedicated thrombolysis team for AIS patients with regular staff training; (2) establishing a fast-track pathway for suspected acute stroke patients and implementing a standardized stroke order set with a dedicated fast-track activation indicator; (3) initiating thrombolytic education immediately upon emergency department admission; (4) using a standardized version for informed consent communication; (5) displaying process reengineering charts and informed consent templates prominently on the wall; (6) ensuring early preparation and distribution of tPA so that thrombolysis can be performed in the CT or emergency room; (7) utilizing a “key performance indicators real-time feedback form” as part of continuous quality improvement.

### 2.4 Outcome measures

The primary outcomes were: (1) the proportion of patients receiving intravenous thrombolysis in the pre- and post-intervention periods; (2) the median DNT before and after the intervention, including changes in median DNT across the four study quarters; and (3) the proportion of patients achieving a DNT < 60 min in the pre- and post-intervention periods. Secondary outcomes included: (1) door-to-glycemic results time; (2) door-to-biochemical results time; (3) door-to-stroke team arrival time (representing the time taken for rapid triage and stroke team notification); (4) informed consent-to-needle time (reflecting the time spent on performing thrombolysis in the CT or emergency room, including early tPA preparation); (5) door-to-CT time; and (6) CT-to-needle time (representing the time spent on standard informed consent procedures).

### 2.5 Statistical analysis

Continuous variables were expressed as mean ± standard deviation or median (interquartile range), and categorical variables as frequencies (percentages). The normality of data was assessed using Shapiro–Wilk test. Differences between groups for continuous variables were analyzed using Student’s *t*-test, analysis of variance (ANOVA), or the Wilcoxon rank-sum test, as appropriate based on data distribution. Categorical variables were compared using the Chi-square test or Fisher’s exact test. The trend of DNT over the study period was assessed using the Cochran–Armitage trend test. Statistical analyses were conducted using IBM SPSS 27.0 software, with a two-tailed *p* value < 0.05 considered statistically significant.

## 3 Results

### 3.1 Baseline characteristics of AIS patients in pre-intervention and post-intervention groups

A total of 35 centers (see Supplement) participated in this study, enrolling 2059 AIS patients (535 in the pre-intervention period and 1,524 in the post-intervention period). The median age was 68 years, and 1,325 (64.4%) were male. The general characteristics of the enrolled patients, stratified by pre-intervention and post-intervention periods, are presented in Table 1. There were no significant differences between the two groups in terms of age (*p* = 0.224) or gender (*p* = 0.123). However, the median (IQR) onset-to-door time differed significantly between the two periods (*p* = 0.020).

**Table 1:** Baseline characteristics of AIS patients in pre-intervention and post-intervention groups.

Characteristics	Pre-intervention (*n* = 535)	Post-intervention (*n* = 1,524)	*p* value
General data			
Age (y ± SD)	66 ± 13	67 ± 13	0.224
Gender (male, %)	359 (67.1)	966 (63.4)	0.123
Weight, kg	65 (60–70)	65 (57–75)	0.318
Medical history			
Diabetes, *n* (%)	121 (22.6)	334 (21.9)	0.737
Hypertension, *n* (%)	325 (60.7)	968 (63.5)	0.254
Dyslipidemia, *n* (%)	43 (8.0)	126 (8.3)	0.867
Smoke			0.027*
Never smoke, *n* (%)	261 (48.8)	854 (56.0)	
Quit smoking, *n* (%)	82 (15.3)	214 (14.0)	
Currently smoking, *n* (%)	181 (33.8)	424 (27.8)	
Uncertain, *n* (%)	11 (2.1)	32 (2.1)	
Alcohol, *n* (%)			0.294
History of alcohol consumption, *n* (%)	162 (30.3)	424 (27.8)	
Never drink, *n* (%)	364 (68.0)	1,060 (69.6)	
Uncertain, *n* (%)	9 (1.7)	40 (2.6)	
Previous ischemic stroke, *n* (%)	108 (20.2)	307 (20.1)	0.983
TIA, *n* (%)	5 (0.9)	13 (0.9)	1.000
Atrial fibrillation, *n* (%)	87 (16.3)	311 (20.4)	0.037*
Myocardial infarction, *n* (%)	14 (2.6)	48 (3.1)	0.535
Clinical characteristics			
Median pre-stroke mRS (IQR)	0 (0–5)	0 (0–5)	0.057
Median NIHSS score at admission (IQR)	9 (4–15)	8 (4–13)	0.016*
Arrival by EMS, *n* (%)	241 (45.0)	510 (33.5)	<0.001***
Systolic pressure at admission, Median, (IQR)	150 (135–170)	150 (134–169)	0.303
Diastolic pressure at admission, Median, (IQR)	85 (75–94)	85 (76–95)	0.623
Onset-to-door time, (min, IQR)	80 (50–120)	90 (55–135)	0.020*

TIA, transient ischemic attack; mRS, modified Rankin Scale; NIHSS, the National Institutes of Health Stroke Scale; IQR, interquartile range; EMS, emergency medical service. *p < 0.05, ***p < 0.001.

### 3.2 Primary outcome analysis

**Figure 3:**
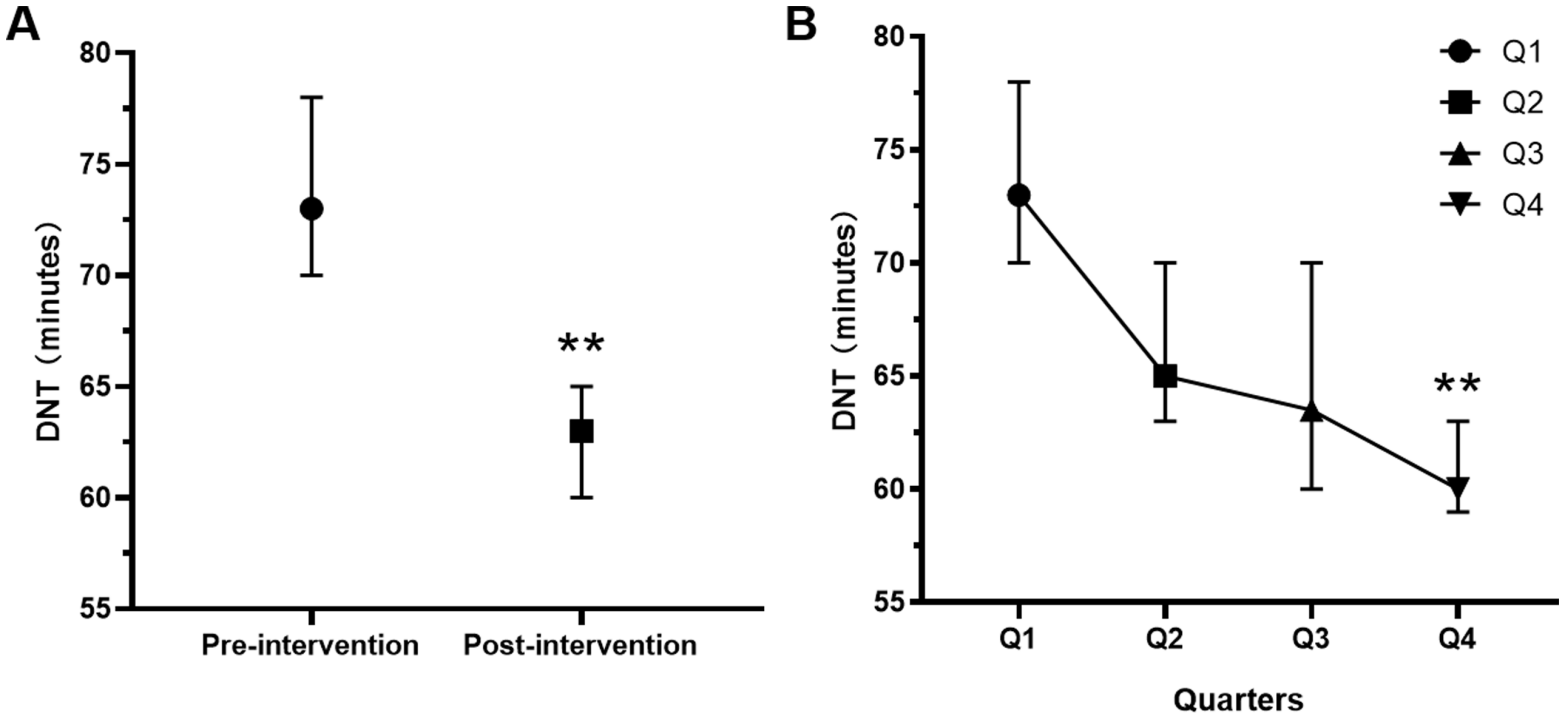
Median DNT with 95% confidence interval for AIS patients with intravenous thrombolysis in the pre-intervention and post-intervention groups **(A)** and different quarters **(B)** (from July 2014 to June 2015, in which 3 months was a quarter. Q1 represents the pre-intervention period, Q2–Q4 represents the post-intervention period). *p* < 0.01; DNT, door-to-need time; AIS, acute ischemic stroke.

**Figure 4:**
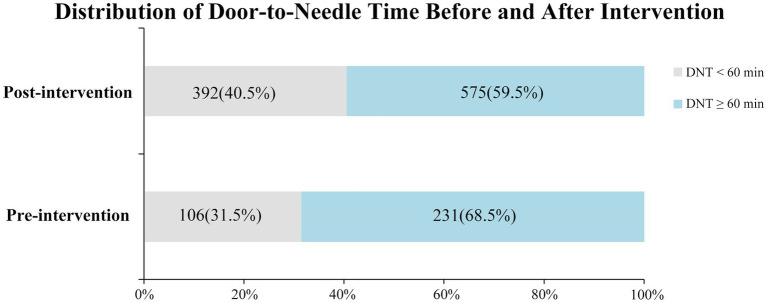
Distribution of door-to-needle time before and after intervention. DNT, door-to-needle time.

After the intervention, the median DNT decreased significantly from 73 min to 63 min (*p* = 0.001) (Figure 3A), demonstrating the effectiveness of the HPR strategy in reducing treatment delays. A consistent downward trend in median DNT was observed compared to the pre-intervention period (P for trend = 0.001; Q4 versus Q1, *p* = 0.002), as shown in Figure 3B. This improvement can likely be attributed to increased familiarity with the reengineering process and the utilization of the ‘Key Performance Indicator Real-Time Feedback Form’ as a tool for continuous quality improvement. Additionally, the proportion of patients with a DNT < 60 min increased significantly from 31.5 to 40.5% after the intervention (*p* = 0.003) (Figure 4). However, there was no significant difference in the proportion of acute ischemic stroke (AIS) patients receiving intravenous thrombolysis between the pre-intervention period (63.0%) and the post-intervention period (63.5%) (*p* = 0.849).

### 3.3 Second outcome analysis

Other time intervals for AIS patients receiving intravenous thrombolysis in the pre-intervention and post-intervention groups were summarized in Table 2. There were no significant differences between the two groups in door-to-glycemic results time (50 min versus 50 min, *p* = 0.464) and door-to-biochemical results time (10 min versus 12 min, *p* = 0.969). However, compared with the pre-intervention group, the median signing informed consent-to-needle time decreased significantly from 10 min (IQR: 5–20 min) to 7 min (IQR: 5–15 min) (*p* < 0.001), and the median CT-to-needle time decreased significantly from 50 min (IQR: 35–72 min) to 43 min (IQR: 32–61 min) (*p* < 0.001). In contrast, door-to-stroke team arrival time (10 min versus 12 min, *p* = 0.256) and door-to-CT time (15 min versus 15 min, *p* = 0.538) did not differ significantly between the two groups.

**Table 2:** Second outcomes.

Time	Pre-intervention (*n* = 337)	Post-intervention (*n* = 967)	*p* value
Door-to-glycemic results time, (min)	20 (5–40)	20 (10–40)	0.464
Door-to-biochemical results time, (min)	50 (36–72)	50 (40–70)	0.969
Door-to-stroke team arriving time, (min)	10 (5–20)	12 (5–23)	0.256
Signing informed consent-to-needle time, (min)	10 (5–20)	7 (5–15)	<0.001***
Door-to-CT time, (min)	15 (10–25)	15 (10–25)	0.538
CT-to-needle time, (min)	50 (35–72)	43 (32–61)	<0.001***

***p < 0.001.

## 4 Discussion

In this multicenter, prospective, nonrandomized quasi-experimental (pre-post) study, the implementation of HPR resulted in a significant reduction of DNT compared with the pre-intervention period. Moreover, continued implementation of HPR led to progressive improvements, as evidenced by a steadily declining DNT and a higher proportion of patients achieving a DNT of less than 60 min, despite the overall intravenous thrombolysis rate remaining unchanged.

Remarkably, our data showed no significant difference in the thrombolysis rate between the pre- and post-intervention periods (63% vs. 63.5%). This rate is approximately six times higher than the 11% reported by the Chinese National Stroke Registry in 2007–2008 (8). This difference may reflect the relatively high level of stroke care capacity and early prioritization of thrombolysis in the participating centers. In contrast, a contemporaneous domestic study applying HPR to patients admitted within 6 h (2016–2019) reported an increase in the thrombolysis rate from 29 to 48% (13). Compared with contemporaneous international data—such as the U. S. GWTG-Stroke study (2012–2013), which reported an 83.6% thrombolysis rate within 3 h (14), our findings suggest a persistent gap between China and developed countries. Importantly, more recent evidence indicates that thrombolysis rates in China have improved substantially. Among patients arriving within the therapeutic time window or meeting eligibility criteria, recent studies report rates of approximately 30–40% (15, 16). Compared with these data, the thrombolysis rate observed in our study appears relatively higher. This difference may be attributable to the higher level of stroke care capacity and resource availability in the participating centers and may also reflect regional disparities in healthcare delivery. Overall, despite these improvements, a gap remains compared with developed countries, highlighting the continued need for process reengineering to further optimize stroke care in China.

Accumulated studies have demonstrated that reducing door-to-needle time (DNT) is crucial for better stroke outcomes (17). In our study, the intervention reduced the median DNT to 63 min—nearly half that reported by CNSR in 2007–2008 (8) and comparable to the “Target: Stroke” study (18)—with the proportion of patients achieving DNT < 60 min rising from 31.5 to 40.5%. This trend suggests that continuous quality feedback can further drive improvements in DNT. The DNT reduction was mainly due to a shortening of the “fragmentation time” across various treatment phases. Notably, the interval from obtaining informed consent to needle insertion was reduced from 10 min to 7 min (*p* < 0.001), highlighting the benefit of the forward-moving thrombolysis protocol. In China, obtaining written informed consent from patients and their families is mandatory for thrombolysis; concerns about bleeding risks, drug costs, and other factors often lead to significant delays. To address this, we introduced a standardized informed conversation process and displayed the corresponding process flowchart to expedite consent. Additionally, treating patients directly in the CT room or emergency department eliminated delays associated with ward transfers, while early discussions between the stroke team and patients or their families further reduced CT-to-needle time. Together, these synergistic measures effectively minimized treatment delays and improved DNT. DNT performance in China has improved substantially in recent years. Contemporary studies report a median DNT of approximately 60 min, with about 53.4% of patients receiving treatment within 60 min (15). In comparison, stroke thrombolysis remains at an early stage in some lower-middle-income settings. For example, early data from Lao PDR reported an overall thrombolysis rate of approximately 5% with a mean DNT of 108 min, whereas a more recent study from Ghana reported a thrombolysis rate of 0.83% and a mean DNT of 2 h and 37 min (19, 20). Our study achieved a median DNT of 63 min through process optimization without relying on costly technological upgrades. These findings suggest that workflow-based interventions may represent a potentially cost-effective and scalable approach in resource-constrained healthcare systems.

Although the present data was collected approximately a decade ago, the findings remain relevant for several reasons. First, this study represents one of the early multicenter, real-world implementations of hospital process reengineering strategies for stroke care in China, providing insight into workflow optimization at a time when standardized stroke systems were not yet fully established. Second, compared with more recent studies that are often conducted within the framework of regional system integration or supported by advanced information technologies, our study focuses specifically on in-hospital process reengineering and organizational optimization, offering an alternative and more pragmatic implementation pathway. Third, in healthcare settings where system-level support remains limited or unevenly developed, such workflow-based interventions may still be highly applicable. Therefore, beyond its historical perspective, this study provides complementary evidence for optimizing stroke care across diverse healthcare contexts.

However, several limitations should be acknowledged. First, the data were collected approximately 10 years ago, which may limit the contemporaneous relevance of the findings. It is important to note that the delay in data collection, which occurred 10 years ago, was due to resource and time limitations within the research team, resulting in an extended analysis and writing process. Additionally, the team faced challenges such as staff turnover and funding shortages during the research period, which further contributed to the delayed publication. Second, this study was non-randomized, which introduces inherent biases such as selection bias and confounding factors. Without random assignment, baseline differences between groups may exist, making it difficult to attribute observed effects solely to the intervention. This limitation is further compounded using a pre-post quasi-experimental design, which compares outcomes before and after the implementation of HPR. Although the pre-intervention period serves as the control group, it is not an independent control group, and the lack of a separate, non-intervened group makes it challenging to rule out the influence of external factors or concurrent changes that could have impacted the results. Additionally, without randomization, confounding variables cannot be fully controlled, limiting the ability to draw strong causal inferences. As a result, causality cannot be definitively established, and the findings may be less robust compared to randomized controlled trials. Finally, the absence of long-term follow-up limits the ability to evaluate the sustained impact of process reengineering strategies on long-term stroke outcomes.

## 5 Conclusion

Hospital process reengineering significantly improved door-to-needle time, highlighting the importance of optimizing workflows in acute stroke care. Although this study was conducted a decade ago, its findings continue to offer valuable insights and practical implications for underdeveloped regions and countries.

## Data Availability

The raw data supporting the conclusions of this article will be made available by the authors, without undue reservation.
