# Supplementary material for: Impact of hospital process reengineering on door-to-needle time for intravenous thrombolysis in acute ischemic stroke (PROMISE-CHINA): a multicenter prospective pre-post quasi-experimental study
Source: Front Neurol. 2026 Apr 10;17:1746553. doi: 10.3389/fneur.2026.1746553 (PMC13105936; doi:10.3389/fneur.2026.1746553)
Supplement: Supplementary file 1 [file Supplementary_file_1.docx]

**PROTOCOL**

Version 1.0 of 2014

| **Impact of Hospital Process Reengineering on Door-to-Needle Time for Intravenous Thrombolysis in Acute Ischemic Stroke (PROMISE-CHINA): A Multicenter Prospective Pre-Post Quasi-Experimental Study** |
| --- |

| **Principle Investigator:**  Anding Xu  Department of Neurology  the First Affiliated Hospital of Jinan University  Guangzhou, 510000, China  Tel: +8613392692160  E-mail: [tlil@jnu.edu.cn](mailto:tlil@jnu.edu.cn) |
| --- |

**Investigator’s Agreement to the Clinical Research Protocol**

As an investigator, I have read the PROMISE-CHINA research protocol and have had a thorough discussion with the sponsor regarding the objectives and content of this clinical research.

I agree to conduct the research in accordance with this clinical trial protocol, abide by its requirements, and adhere to ethical and safety standards.

I understand that if the sponsor decides, for any reason, to terminate or suspend the clinical research at any time, I will be notified in writing. Similarly, if I decide to withdraw from the clinical trial, I will notify the sponsor in writing.

**Investigator:**

**Institution Name:** __________________________________
**Institution Address:** _________________________________
**Postal Code:** ______________________________________
**Contact Number:** __________________________________
**Name:** _________________________________ (Signature)
**Date of Signing:** __________________________________

**Table of Contents**

[Abstract 4](#_Toc193030324)

[1. Background and current state of knowledge 6](#_Toc193030325)

[1.1 Current Status of Intravenous Thrombolysis for AIS in China 6](#_Toc193030326)

[1.2 Factors Hindering Intravenous Thrombolysis for AIS in China and Improvement Experience Domestically and Abroad 6](#_Toc193030327)

[1.3 Process Reengineering and Improvement of Medical Quality 9](#_Toc193030328)

[Reference 13](#_Toc193030329)

[2. Research Objective: 16](#_Toc193030330)

[3. Study Design 16](#_Toc193030331)

[3.1 Protocol Description 16](#_Toc193030332)

[3.2 PROMISE-China Study Organization Committee 16](#_Toc193030333)

[3.3 Study Center Inclusion and Exclusion Criteria 17](#_Toc193030334)

[3.4 In-hospital Intravenous rt-PA Thrombolysis Process Diagnosis and New Process Design 17](#_Toc193030335)

[3.5 Study Phases 21](#_Toc193030336)

[4. Patients Enrolled in the Study 21](#_Toc193030337)

[4.1 Sample Size 21](#_Toc193030338)

[4.2 Inclusion criteria 21](#_Toc193030339)

[4.3 Exclusion criteria 22](#_Toc193030340)

[4.4 Treatment Plan for Patients Enrolled in This Study 22](#_Toc193030341)

[5. Study Endpoints 22](#_Toc193030342)

[5.1 Primary outcome: 22](#_Toc193030343)

[5.2 Secondary outcome: 22](#_Toc193030344)

[5.3 Efficacy and safety outcome 22](#_Toc193030345)

[5.4 Prespecified Additional Endpoints and Analyses 23](#_Toc193030346)

[6. Study Withdrawal 23](#_Toc193030347)

[6.1 Withdrawal of Study Centers 23](#_Toc193030348)

[6.2 Handling of Patient Withdrawal from the Registry 23](#_Toc193030349)

[6.3 Handling of Patients Who Refuse to Join the Study 23](#_Toc193030350)

[7. Study Follow-Up 24](#_Toc193030351)

[8 Data Management and Statistical Analysis 24](#_Toc193030352)

[9. Modification of the Clinical Trial Protocol 25](#_Toc193030353)

[10. Study Monitoring 25](#_Toc193030354)

[11. Confidentiality 25](#_Toc193030355)

[12. Ethics and Regulations 25](#_Toc193030356)

[13. Early Termination of the Study or Early Closure of Study Centers 26](#_Toc193030357)

[14 Clinical Research Results, Research Intellectual Property, and Publication 27](#_Toc193030358)

[Appendix Directory: 28](#_Toc193030359)

Abstract

| **Study ID** | 2011BAI08B02-02 |
| --- | --- |
| **Title** | Impact of Hospital Process Reengineering on Door-to-Needle Time for Intravenous Thrombolysis in Acute Ischemic Stroke (PROMISE-CHINA): A Multicenter Prospective Pre-Post Quasi-Experimental Study |
| **Principle Center** | The First Affiliated Hospital of Jinan University |
| **Objective** | Based on Business Process Reengineering (BPR) theory from enterprise management, the intravenous thrombolysis process for Acute Ischemic Stroke in hospitals will be reengineered to increase the rate of intravenous thrombolysis among patients arriving within 3.5 hours from symptom onset, and to shorten the door-to-needle time (DNT) from patient arrival at the emergency department to the start of thrombolysis. |
| **Efficacy Outcomes** | Primary outcome:   1. The median DNT before and after the intervention, as well as changes in median DNT across the four study periods 2. The proportion of patients achieving DNT <60min during the pre- and post-intervention periods 3. The proportion of intravenous thrombolysis in the pre- and post-intervention periods.   Secondary outcome:   1. Door-to-glycemic results time. 2. Door-to-biochemical results time. 3. Door-to-stroke team arrival time, representing the time spent on rapid triage and stroke team notification 4. Informed consent-to-needle time 5. Door-to-CT time 6. CT-to-needle time |
| **Trial Design** | PROMISE-CHINA is multicenter, prospective, nonrandomized quasi-experimental (pre-post) study to evaluate the impact of the reengineered strategy on the proportion of eligible patients receiving IV-tPA treatment and door-to-needle time compared with those receiving standard medical care and hospital processes. |
| **Trial Population** | Patients diagnosed with AIS presenting within 3.5 hours of symptom onset. |
| **Sample Size** | 2000 |
| **Inclusion criteria** | 1. Patients diagnosed with AIS presenting within 3.5 hours of symptom onset. 2. Written informed consent obtained from the patient or a legally authorized representative. |
| **Exclusion criteria** | 1. Patients with complete resolution of neurological deficits, consistent with transient ischemic attack. 2. Patients with radiologically confirmed silent cerebral infarctions lacking clinical manifestations. 3. Patients with contraindications to intravenous thrombolytic therapy. |
| **Intervention Methods:** | 1. Apply BPR theory, analyze domestic and international research, and propose a basic framework for reengineering the intravenous thrombolysis process in medical centers suitable to the national context. 2. The study period is 12 months, with the first 3 months serving as the baseline period. Each center formulates a new process based on the basic process reengineering framework, begins gradual implementation in the fourth month, and continuously refines the process reengineering plan. 3. The basic process reengineering framework will be delivered as a comprehensive intervention package that outlines uniform key reengineering elements, provides a standardized series of tools for process management, and establishes a real-time monitoring and feedback system for core quality indicators. 4. The standardized series of tools supporting process reengineering include: a rapid identification tool for suspected stroke by emergency department personnel; a stroke team construction plan; a real-time feedback form for key early-stage quality control indicators in ischemic stroke; a standardized thrombolysis informed consent form and public service announcement posters for informed discussions; recommended indications and contraindications for intravenous thrombolysis; and a standardized operating procedure (SOP) for intravenous rt-PA thrombolysis, among others. 5. Continuous improvement measures: Use the “Real-time Feedback Form for Key Early-Stage Quality Control Indicators in Ischemic Stroke” to monitor and provide feedback on the reengineered process; the study committee will summarize the research results every three months to further refine the process reengineering plan. |
| **Statistical Analysis** | Continuous variables were expressed as the mean ± standard deviation or median (interquartile quartiles), while categorical variables were presented as frequencies (percentage). Differences between groups in continuous variables were assessed using Student’s t-test, analysis of variance (ANOVA) or Wilcoxon rank-sum test, depending on the distribution of the data. Categorical variables were compared using the Chi-square test or Fisher’s exact test. Statistical analyses were conducted using IBM SPSS 27.0 software, and a 2-tailed P value of < 0.05 was considered statistically significant. |
| **Sites Number** | 35 |
| **Duration** | 12 months |

1. Background and current state of knowledge

Intravenous thrombolysis using recombinant tissue-type plasminogen activator (rt-PA) within 4.5 hours from symptom onset is the only treatment that can reduce disability in acute ischemic stroke (AIS). Both domestic and international guidelines or consensus statements uniformly and strongly recommend the use of intravenous rt-PA thrombolysis within 4.5 hours of AIS onset; moreover, the earlier the thrombolysis is performed, the greater the benefit and the lower the risk [1, 2]. Based on the results of China’s “Ninth Five-Year” key research project, intravenous urokinase thrombolysis has been recommended by the Chinese Cerebrovascular Disease Guidelines (applicable within 6 hours of onset); however, it has not been recognized by international peers. The 2013 American guidelines explicitly recommend that intravenous urokinase thrombolysis be limited to use in clinical research [2]. Although endovascular treatments, including intra-arterial thrombolysis, may be applicable to some patients with large vessel occlusion, there is no evidence that they are superior to intravenous rt-PA thrombolysis. Currently, guidelines recommend that patients eligible for intravenous thrombolysis within 4.5 hours of onset should preferentially receive intravenous rt-PA [2], a recommendation further supported by three large randomized controlled trials published in 2013 [3].

1.1 Current Status of Intravenous Thrombolysis for AIS in China

Although intravenous rt-PA thrombolysis within the designated time window is the most effective and recommended treatment for AIS, its global utilization remains very low due to time window limitations and other factors. Recent reports from the United States indicate that it is administered in only 2.4%–5.2% of all AIS patients [4]. Moreover, even among those arriving within the thrombolysis window, the proportion receiving thrombolysis is not high; a recent study in Australia showed that among AIS patients arriving at the hospital within 4.5 hours of onset, only 14.7% received thrombolysis [5]. The situation in China is even more concerning. Three multicenter studies in China have shown that intravenous thrombolysis is performed in less than 2% of all AIS patients, and the proportion of patients receiving intravenous rt-PA thrombolysis within 3 hours of onset is only 7.2%–8.9% [6, 7] In contrast, in U.S. hospitals following guidelines, the intravenous rt-PA thrombolysis rate has increased to 70% among AIS patients arriving at the emergency department within 2 hours of onset [8].In addition to the overall low thrombolysis rate, particularly the low rate among patients arriving within the time window, AIS thrombolysis in China has the following characteristics: The use of low-dose rt-PA for intravenous thrombolysis. In a multicenter cross-sectional study across seven cities, 13 out of 15 patients receiving intravenous rt-PA thrombolysis were treated with a low dose; the CNSR study showed that 44% of patients receiving intravenous rt-PA thrombolysis were administered a low dose [9].Some patients receive intravenous urokinase thrombolysis, and a small number of patients receive intravenous rt-PA beyond the 4.5-hour window. Furthermore, some patients who meet the criteria for intravenous thrombolysis are recommended to receive intra-arterial thrombolysis, and the indications for endovascular interventions are sometimes indiscriminately expanded, although specific data from nationwide or multicenter studies are lacking.

1.2 Factors Hindering Intravenous Thrombolysis for AIS in China and Improvement Experience Domestically and Abroad

1.2.1 Pre-hospital Factors

Consistent with international findings, the most important reason for the low thrombolysis rate in AIS is that patients fail to reach a thrombolysis-capable hospital within the time window, thus missing the opportunity for thrombolysis. Data from three multicenter studies in China show that approximately 80% of AIS patients do not arrive at the hospital within 3 hours from symptom onset [6, 7], which is not significantly different from data in developed countries abroad [5, 10]. The primary reason for patients failing to arrive within the time window, both domestically and internationally, is the public’s insufficient knowledge of stroke (early signs, appropriate responses), leading to failure to call the emergency medical service (EMS) or seek timely medical care [11-13].

Furthermore, foreign studies have shown that seeking help without calling EMS prolongs treatment times and reduces the likelihood of thrombolysis [10, 14, 15]. In recent years, in the United States the proportion of acute or subacute stroke patients arriving at hospitals without EMS remains as high as 50% [15], whereas data from the CNSR study in China indicate that this proportion is as high as 80% [7].

Both domestic and international studies have demonstrated that public health education can significantly improve awareness of early stroke symptoms and the proper measures to take after stroke, increase the proportion of EMS calls, markedly reduce pre-hospital delays, and raise thrombolysis rates. However, once the intervention ceases, the rates return to baseline[4, 15, 16]. Simple, practical, and highly sensitive health education tools represented by the FAST score have proven effective [2, 15, 17]. “FAST” stands for Facial drooping, Arm weakness, Speech difficulties, and Time (to call EMS); when one or more of these signs are present, an immediate call to EMS is warranted.

Another important factor contributing to pre-hospital delay is that suspected stroke patients are taken to hospitals that do not have thrombolysis capabilities [4, 15, 17]. A Swiss study showed that 20% of AIS patients within 3 hours of onset were transported to hospitals without thrombolysis capability [18]. Insufficient stroke recognition by emergency personnel, failure to quickly and correctly identify stroke patients, and not prioritizing suspected stroke patients (as in Acute Stroke Ready Hospitals, ASRH) further contribute to delays [4, 15].

Lack of awareness about the AIS thrombolysis time window and transporting patients to hospitals that do not perform thrombolysis are the main causes of delay. Targeted stroke training for EMS personnel can significantly reduce transportation-related delays [4, 15, 17]. The effectiveness of rapid stroke identification tools for EMS personnel—such as FAST, the Cincinnati Prehospital Stroke Scale, and the Los Angeles Prehospital Stroke Scale—has been confirmed [4, 10, 14, 15]. In the United States, the certification of stroke centers and Acute Stroke Ready Hospitals (ASRH) provides a basis for EMS to preferentially transport stroke patients to appropriate hospitals; in some regions, laws or administrative measures stipulate that suspected stroke patients can only be transported to stroke centers. In contrast, China currently lacks a systematic stroke training program for EMS personnel and has not implemented a certification system for stroke centers or similar ASRH programs. Moreover, not all hospitals—even tertiary hospitals—perform AIS thrombolysis. Consequently, in China the phenomenon of EMS transporting suspected stroke patients to hospitals without thrombolysis capabilities may be even more severe. A 2007 survey found that among 48 tertiary hospitals and 76 secondary hospitals in Beijing, only 54 facilities carried out intravenous rt-PA or urokinase thrombolysis [19]. Another characteristic in China is that the management systems of EMS personnel and ambulances, which are under the control of the local “120” command system, vary across regions. Under current conditions, lacking stroke center or ASRH certification and systematic EMS training, and with hospitals operating under a self-sustaining model—the ambulance teams tend to prioritize transporting patients back to their affiliated centers rather than to hospitals capable of thrombolysis.

1.2.2 In-hospital Factors

In-hospital delay is an important factor hindering thrombolysis in AIS patients, particularly for those arriving at the emergency department within the time window. The time from arrival at the emergency department to the administration of intravenous rt-PA (door-to-needle time, DNT) is an indicator of in-hospital delay, involving multiple departments and processes after admission. Although some in-hospital delay is inevitable, the DNT without process interventions generally far exceeds 60 minutes. For instance, data from a seven-city study in China showed an average DNT as high as 167 minutes, and during the 11th Five-Year Plan period, the Chinese National Stroke Registry (CNSR) study reported an average DNT of 115 minutes, with the proportion of patients achieving DNT <60 minutes being less than 10%—a huge gap compared with contemporaneous figures from the United States and Canada [7]. Prolonged DNT results in some patients who could have received thrombolysis within the time window, losing the opportunity for treatment. The main reasons for the extended DNT include [4, 5, 15, 19]:(1) The emergency department’s inability to rapidly and correctly assess patients and implement simple interventions, reflecting insufficient understanding of stroke symptoms, stroke treatment, and the time-dependent nature of thrombolysis;(2) An inadequate acute stroke management system, such as the lack of an on-call stroke team, the absence of an emergency clinical pathway for rapid neurological imaging and necessary laboratory tests for thrombolysis-eligible patients;(3) Unnecessary multimodal imaging examinations;(4) Uncertainty among stroke team physicians regarding the implementation of thrombolysis. It should be emphasized that the evidence for the benefit of intravenous rt-PA thrombolysis is based on studies that screened patients using clinical assessment and plain CT scans, and multimodal imaging examinations can significantly prolong the DNT [23]. Current guidelines clearly recommend that multimodal imaging should not lead to prolonged in-hospital delays[1, 2, 15]. Another significant factor in prolonging DNT is that unlike in developed countries such as the United Kingdom and the United States where informed consent is not required for intravenous rt-PA thrombolysis, in China the process requires a signed written informed consent. Due to the relatively limited knowledge about stroke and thrombolysis among patients and their families, combined with a lack of trust in medical personnel, conflicts between doctors and patients are prominent. This often leads to failures in the informed consent conversation or significant delays in obtaining consent, further reducing the thrombolysis rate among eligible patients and extending the DNT. CNSR data indicate that the average time from obtaining the imaging results to administering the thrombolytic agent is 85 minutes—30 to 60 minutes longer than in the United States or Europe. A series of studies in developed countries have shown that monitoring and optimizing the in-hospital thrombolysis process can significantly shorten DNT, improve the effectiveness of thrombolysis, and reduce the risk of hemorrhage [8, 15, 20, 21]. For example, the US "Get With The Guidelines" program demonstrated that the proportion of patients with DNT <60 minutes has increased to 30%, and the subsequent TARGET study, initiated in 2010 to further shorten DNT, showed a marked inflection point with the proportion of DNT <60 minutes rising from 29.6% to 54.2%, with annual increases before and after the program of 1.32% versus 6.24% respectively [22, 23]. According to studies from developed countries in Europe and the United States, the main reasons for not administering thrombolysis to patients arriving within the time window include: age >80 years; mild stroke or rapid improvement of symptoms before thrombolysis; and physicians’ perception of contraindications for thrombolysis (including NIHSS >25, extensive early CT signs of infarction, or the presence of low-density lesions). Data from a seven-city study in China indicated that the reasons for not administering thrombolysis were as follows: age (>80 years or <18 years) (28.9%), stroke symptoms being too mild (24.0%), rapid recovery (16.5%), CT already showing infarct lesions (15.7%), time >3 hours (15.7%), and stroke symptoms being too severe (7.4%), with patient/family refusal accounting for 18.2%. A single-center study reported that the most important factors were NIHSS score <5 or rapid improvement of symptoms before thrombolysis (44 out of 60, 73%), followed by patient/family refusal at approximately 20%. In addition, some domestic research centers believe that cardioembolic stroke, represented by atrial fibrillation, is not suitable for thrombolysis; however, specific data on the number of patients not receiving thrombolysis for this reason are lacking.

1.3 Process Reengineering and Improvement of Medical Quality

1.3.1 Process Reengineering (Business Process Reengineering, BPR)

BPR was first proposed in the early 1990s by Michael Hammer, a professor at MIT, and James Champy, Chairman of CSC Management Consulting. BPR is defined as: "In order to achieve a quantum improvement in the fundamental operating dimensions of modern enterprises—such as cost, quality, service, and speed—it is necessary to fundamentally rethink and completely redesign the work processes." BPR emphasizes taking business processes as the object and center of transformation, focusing on customer needs and satisfaction as the goal, and completely changing the traditional work methods that, since the Industrial Revolution, have divided a complete task into separate parts to be performed sequentially by relatively independent departments. It involves a radical rethinking and thorough redesign of the existing business processes, using advanced manufacturing technologies, information technology, as well as dramatic improvements in modern management principles, quality, service, and speed.

1.3.2 Application of BPR in Clinical Medicine and Intravenous Thrombolysis for AIS

After the introduction of BPR theory, its application was mainly seen in hospital management [23, 24][30-31], and there have been similar reports of BPR application in hospital management in China. In clinical medicine, the application of BPR has been primarily focused on the management of emergency department workflows or surgical management. In 2010, the American College of Emergency Physicians published a white paper on using BPR to improve emergency services [25], and there are also a few clinical reports demonstrating significant improvements in emergency department processes after BPR interventions [26]. Abroad, studies have begun to apply theories from business management to improve the quality of AIS thrombolysis. For example, a European study showed that the application of the Toyota Lean principles can significantly shorten the DNT for AIS patients [21]. In a dedicated study aimed at shortening DNT, the U.S. TARGET expert group extracted ten “best practice strategies” (Table 1) from previous global research that were rapid to implement, feasible, and economically valuable as measures to improve DNT, and these served as guiding measures for centers participating in the TARGET study [22]. A recently published result from another TARGET research group indicated that among the ten measures, rapid triage/stroke team activation, a single-call activation system, and storing rt-PA in the emergency department were three independent factors that could significantly shorten DNT; other measures that contributed to shortening DNT included: a 24/7 on-call stroke team, training of primary medical staff, an intravenous rt-PA operation manual with pre-mixing of rt-PA, and regular feedback on DNT time indicators [27].

| Strategy | Best Practice | Explanation |
| --- | --- | --- |
| 01 | Advance hospital notification by EMS | EMS providers should, if feasible, provide early notification to the receiving hospital when stroke is recognized in the field. Advance notification of patient arrival by EMS can shorten time to CT and improve the timeliness of treatment with thrombolysis. |
| 02 | Rapid triage protocol and stroke team notification | Acute triage protocols facilitate the timely recognition of stroke and reduce time to treatment. A single-call activation system for the stroke team should be activated as soon as the stroke patient is identified in the emergency department or after notification from pre-hospital personnel. |
| 03 | Single-call activation system | A single-call should activate the entire stroke team. A single-call activation system for the stroke team in which the emergency department calls a central page operator, who then simultaneously pages the entire stroke team, including notification for stroke protocol initiation. |
| 04 | Stroke tools | A stroke toolkit containing clinical decision support, stroke-specific guides, hospital-specific algorithms, critical care pathways, NIH Stroke Scale, and other stroke tools should be available and updated. |
| 05 | Rapid acquisition and interpretation of brain imaging | It is essential to initiate a CT scan (or MRI) within 25 min of arrival and complete interpretation of the CT scan within 45 min of arrival to exclude intracranial hemorrhage prior to administration of thrombolytic therapy. |
| 06 | Rapid laboratory testing (including point-of-care testing if indicated) | When indicated, laboratories such as platelet count—and for patients in whom coagulation parameters should be assessed due to suspicion of coagulopathy—INR (International Normalized Ratio)/PTT results should be available as quickly as possible and no later than 45 min after arrival. If standard laboratory turnaround times cannot meet this target, point-of-care testing in the emergency department should be used to generate the needed timeframe. |
| 07 | Mix tPA medication and administer bolus dose | Mix drug and set up the bolus dose and 1-hour infusion pump as soon as a patient is recognized as a possible tPA candidate, even before brain imaging. Early preparation allows tPA infusion to begin as soon as the medical decision to treat is made. This minimizes delays for patients who are not excluded from tPA therapy and helps ensure that the drug is administered at the most appropriate time window in the acute setting. |
| 08 | Rapid access to intravenous tPA | Once eligibility has been determined and intracranial hemorrhage has been excluded, intravenous tPA should be promptly administered. tPA should be readily available in the emergency department or in the pharmacy and accessible for immediate administration following physician approval. |
| 09 | Team-based approach | The team approach based on standardized stroke pathways and protocols has proven to be effective in increasing the number of eligible patients treated and reducing time to treatment in stroke. An interdisciplinary collaborative team is essential for optimal stroke care performance to deliver timely administration and minimize dosing errors. |
| 10 | Prompt data feedback | Accurately measuring and tracking your hospital’s processes (door-to-needle times, IV tPA treatment rates) can lead to meaningful improvements in care and outcomes. Data feedback should occur at regular intervals (ideally monthly) with a direct link to continuous improvement efforts and should include performance measures, both process and outcomes, based on data collection. |

EMS indicates emergency medical system; CT, computed tomography; MRI, magnetic resonance imaging; INR, International Normalized Ratio; PTT, Prothrombin Time; IV, intravenous; tPA, tissue-type plasminogen activator; MRI, magnetic resonance imaging; IV tPA, intravenous tissue-type plasminogen activator.

Table 1. Ten best practice strategies

1.3.3 Analysis of the Current Situation of AIS in China Using BPR Theory
In summary, compared with developed countries abroad, the use of intravenous thrombolysis for treating AIS in China is significantly lower, and the gap is even wider within the time window. Moreover, both pre-hospital and in-hospital delays are longer. Advanced international experiences suggest that targeted management interventions can improve thrombolysis rates, its effectiveness, and safety. Some of these experiences are derived from the application of advanced business management theories, focusing on the optimization and reengineering of in-hospital processes. However, the current situation of AIS thrombolysis in China is not entirely consistent with that abroad—it is more complex and cannot simply copy foreign experiences. It is essential to establish a management system that is suitable for China's conditions and has universal application value.

The National “Twelfth Five-Year” Science and Technology Support Project, “Research on the Standardized Application of Acute Phase Diagnosis and Treatment Technology for Cerebrovascular Diseases and the Evaluation and Continuous Improvement of Medical Quality” (2011BAI08B02), undertaken by Beijing Tiantan Hospital, Capital Medical University (known as the “Jinqiao Project”), has been fully launched. Phase I data collection has been completed, and Phase II interventional research is about to commence. The Jinqiao Project includes quality improvement indicators for intravenous rt-PA thrombolysis, with two clear targets: increasing the intravenous rt-PA thrombolysis rate for patients arriving within 2 hours to over 20% and achieving a DNT (<60 minutes) proportion of over 20%. In addition, the project also covers other acute-phase quality control indicators beyond thrombolysis. Based on the quality management theory PDSA (Plan, Do, Study, Act) recommended by U.S. quality management expert Dr. W. Edwards Deming, the Jinqiao Project adopts continuous quality improvement measures.

To further improve the quality of intravenous thrombolysis for AIS in China, the Jinqiao Working Group decided to establish a special project titled “Establishment of an Acute-Phase Thrombolytic Treatment Improvement System for Ischemic Stroke” (original English name: Multidisciplinary and Organized Stroke Thrombolytic Therapy Project, MOST).
Beginning in January 2013, the MOST project team, using the PDSA framework as its core, consecutively conducted preliminary surveys at more than 30 centers in Beijing, Shanghai, and Guangzhou. These preliminary studies showed a certain promoting effect on intravenous rt-PA thrombolysis (with an increase in the national usage of rt-PA by neurology departments). However, due to incomplete data reporting (severe underreporting of non-thrombolysis cases and serious logical errors in DNT data), the project team decided to suspend the MOST pilot study and adjust the research protocol and plan before commencing formal research.

After summarizing and analyzing the characteristics of AIS intravenous thrombolysis in China, the advanced experiences abroad, and lessons from the MOST pilot study, the expert group for “Establishment of an Acute-Phase Thrombolytic Treatment Improvement System for Ischemic Stroke” concluded that, in order to achieve the two major targets proposed by the Jinqiao Project, the current focus should mainly be on reengineering the thrombolysis process at medical centers. This is because interventions targeting pre-hospital non-hospital factors are difficult and less feasible. Two main aspects are highlighted:

1. **Health Education:**
   China’s vast population and the widespread presence of rural and underdeveloped areas make public stroke health education a formidable task. The requirement for routine education further adds to the challenge of long-term persistence. It requires government leadership, long-term financial investment from the government, and organized implementation by professionals, with active participation from various medical institutions, media, non-governmental, and charitable organizations. Although hospitals, stroke team members, and other medical personnel are the key forces in the actual implementation, their capabilities and time are limited. Without support from the government, financial resources, and media, achieving significant results in the short term is difficult.
2. **Pre-hospital Transport:**
   Due to the lack of stroke centers or a certification system similar to the U.S. ASRH, as well as the diversity of EMS systems, breakthrough progress in pre-hospital delays cannot be expected in the short term. Only after the emergency teams of medical centers that have already implemented AIS thrombolysis receive EMS calls can corresponding process reengineering work be initiated.

Correspondingly, reducing in-hospital delays and increasing the rate of intravenous rt-PA thrombolysis for patients arriving within the time window are tasks that all medical centers can and should strive to achieve. Applying BPR theory to analyze the characteristics of in-hospital delays in China shows that intravenous rt-PA thrombolysis plays a dominant role. There are prominent and unique features in China that differ from developed countries, such as the need for informed consent discussions; prolonged DNT, especially the extended time from imaging results to thrombolysis; and interference from the use of intravenous urokinase thrombolysis. These factors prevent the direct adoption of advanced foreign experiences. Traditional hospital and stroke management in China is based on a single management system from the emergency department to the neurology team. There is a need to reengineer in-hospital process management using BPR to shift to a patient-centered, disease-centered process management. This requires multidisciplinary reengineering of the AIS thrombolysis process within hospitals. Based on evidence-based medicine and guideline recommendations, the reengineering of the thrombolysis process should be dominated by intravenous rt-PA. Therefore, based on literature analysis, domestic and international experience, and lessons learned from the MOST pilot study, the expert group for “Establishment of an Acute-Phase Thrombolytic Treatment Improvement System for Ischemic Stroke” has developed a hospital process reengineering plan for intravenous thrombolysis treatment of AIS that is suitable for China and feasible. This plan aims to improve the intravenous rt-PA thrombolysis rate within the time window and shorten DNT, which is the subject of the current study titled **“**Hospital Processes Reengineering of Intravenous Thrombolysis in Acute Ischemic Stroke in China: PROMISE-CHINA.”

PROMISE-CHINA is designed to explore whether this in-hospital process reengineering plan is suitable for Chinese medical centers and to determine its effect on increasing the thrombolysis rate for AIS patients arriving within the time window and shortening DNT.

2. Research Objective:
Based on business management BPR theory, propose a hospital AIS intravenous rt-PA thrombolysis process reengineering plan that is feasible and tailored to China’s national conditions. The plan aims to significantly increase the intravenous rt-PA thrombolysis rate for AIS patients arriving within 3.5 hours (to over 20%, representing an increase of more than 40% compared to baseline) and significantly raise the proportion of cases with a door-to-needle time (DNT) of less than 60 minutes (to over 20%, with an increase of more than 40% compared to baseline). Simultaneously, during the project implementation, the effectiveness and safety indicators of intravenous rt-PA thrombolysis for AIS should reach levels consistent with those in developed countries in Europe and the United States.

3. Study Design

3.1 Protocol Description
This is a nationwide, multicenter, prospective, longitudinal, non-randomized cohort study focusing on reengineering the thrombolysis process at the participating centers.

3.2 PROMISE-China Study Organization Committee

- **Principal Investigators:**

Xu Anding, The First Affiliated Hospital of Jinan University

Wang Yongjun, Beijing Tiantan Hospital, Capital Medical University

- **Study Steering Committee:**

Wang Yongjun (Chair, Beijing Tiantan Hospital, Capital Medical University)

Dong Qiang (Huashan Hospital, Fudan University)

Zeng Jinsheng (The First Affiliated Hospital of Sun Yat-sen University)

Li Yansheng (Renji Hospital, Shanghai Jiao Tong University)

Xu Anding (The First Affiliated Hospital of Jinan University)

Xu Yun (Nanjing Drum Tower Hospital)

David Wang (Stroke Center, Illinois State University, USA)

Wang Shaoshi (Branch of Shanghai First People’s Hospital)

Wang Xin (Zhongshan Hospital, Fudan University)

Zhao Gang (Xijing Hospital, Fourth Military Medical University)

- **Study Executive Committee:**

Xu Anding (Chair)

Wang Yilong (Beijing Tiantan Hospital, Capital Medical University)

Huang Lian (The First Affiliated Hospital of Jinan University)

Zhang Yusheng (The First Affiliated Hospital of Jinan University)

Wang Chunjuan (Beijing Tiantan Hospital, Capital Medical University)

Heads of each participating center.

**Executive Committee Secretary:** Li Mu (The First Affiliated Hospital of Jinan University).

- **Data Monitoring Committee:**
  An independent third-party statistical expert panel is appointed to form the independent Data Monitoring Committee. Their main responsibilities include providing constructive feedback on the study protocol, drafting the statistical analysis plan in accordance with the protocol, designing the study, summarizing and registering data from each center to establish a database, and performing statistical analyses and preparing the statistical report according to the plan.
- **3-Month Follow-up Working Group:**
  Led by Huang Lian, with members Zhang Yusheng, Li Mu, Zhao Ying, Dong Dawei, and Cai Yeyan. The study adopts a unified, blinded follow-up of the enrolled patients’ 3-month clinical endpoints.
- **Safety Monitoring Committee:**
  Chaired by Liu Liping. This committee is responsible for conducting periodic safety monitoring of the thrombolysis data from each participating center.
- **Clinical Research Protocol Formulation Committee:**
  Chaired by Xu Anding, with members including Wang Yongjun, Wang Yilong, Wang Chunjuan, and heads of each participating center.

3.3 Study Center Inclusion and Exclusion Criteria

**Inclusion Criteria**

1. Patients diagnosed with AIS presenting within 3.5 hours of symptom onset.
2. Written informed consent obtained from the patient or a legally authorized representative.

**Exclusion Criteria**

1. Patients with complete resolution of neurological deficits, consistent with transient ischemic attack.
2. Patients with radiologically confirmed silent cerebral infarctions lacking clinical manifestations.
3. Patients with contraindications to intravenous thrombolytic therapy.

3.4 In-hospital Intravenous rt-PA Thrombolysis Process Diagnosis and New Process Design

3.4.1 Process Diagnosis and New Process Design
Based on BPR theory, an analysis of the results of three previous large-scale multicenter studies in China, along with an examination of domestic hospital management processes, has led to the identification of the following key factors that hinder in-hospital thrombolysis and the corresponding solutions for a new process. Please refer to Table 2 and Figure 2.

**Table 2: In-Hospital IV rt-PA Thrombolysis Process Diagnosis and Reengineering Plan for Chinese AIS Patients**

| **Key Factors** | **Main Reasons** | **Process Reengineering and Supporting Measures** |
| --- | --- | --- |
| **EMS—Stroke Team Admission Delay** | Lack of stroke center certification, diverse EMS management in China, EMS personnel lacking rapid stroke recognition knowledge, and lack of an effective mechanism to activate a thrombolysis-capable medical center | 1. EMS management mechanism reform / stroke center certification: requires government departments, cannot be accomplished by hospitals  2. Provide FAST tool training, etc. to the hospital emergency teams participating in EMS dispatch, so they can directly activate the in-hospital stroke team |
| **Delay from ED Admission to Stroke Team Member Admission** | 1. ED staff lacking rapid stroke recognition knowledge  2. ED → ED imaging, ED laboratory → re-triage to neurology team → stroke team member deciding whether to administer thrombolysis | 1. Train ED staff using FAST tool, etc.  2. Establish a process for the ED to directly activate the stroke team (arrival to stroke team member <15 minutes). Stroke team member refers to a physician authorized to make thrombolysis decisions |
| **Delay from ED to Obtaining ED Imaging Results** | 1. Lack of an in-hospital stroke “green channel”  2. Lack of a priority imaging process for acute stroke  Blindly expanded multimodal imaging  3. Waiting for a written radiologist report | 1. Rebuild processes giving priority to in-hospital acute stroke patients  2. After rapid identification of suspected stroke, the ED team member directly activates the imaging team  3. Standardize imaging protocols for acute stroke  Stroke team members interpret imaging results |
| **Delay from ED to Obtaining ED Laboratory Results** | 1. Blindly waiting for blood tests (CBC, coagulation profile, blood chemistry, etc.)  2. Lack of an in-hospital “green channel”  3. Lack of a priority laboratory process for acute stroke | 1. According to the latest U.S. guidelines, simplify the mandatory tests for most patients  2. Establish a priority laboratory process for acute stroke patients |
| **Delay from Obtaining Imaging Results to Thrombolysis** | 1. Waiting for informed consent discussion  2. Completing hospital admission procedures, transferring to the stroke unit (ward), and only then starting thrombolysis | 1. While preparing for imaging, a stroke team physician follows the patient to the CT scan and initiates the informed consent discussion  2. Standardize informed consent operating procedures and tools  Post thrombolysis health education posters in ED, radiology  3. Move the “battlefield” for thrombolysis to the ED or imaging department  4. Deliver thrombolytic drugs and related materials to the imaging center or ED |
| **Other Factors Hindering Thrombolysis** | 1. Patient/family refusal of thrombolysis  2. Concerns about thrombolysis in elderly patients, mild stroke, rapid improvement, cardioembolic stroke, or early infarct signs  3. Blindly using endovascular therapy | 1. Implement the above scientific, standardized informed consent process  2. Establish standardized thrombolysis indications and contraindications to increase the thrombolysis rate in eligible patients  3. Provide stroke team member training |
| **Others** | Low-dose rt-PA  Intravenous urokinase thrombolysis | 1. Stroke team member stroke training  2. Establish standardized thrombolysis operating procedures |
| **Continuous Improvement** | Lack of monitoring, feedback, and improvement measures | Use real-time monitoring and feedback forms for key time indicators to achieve continuous improvement |

A


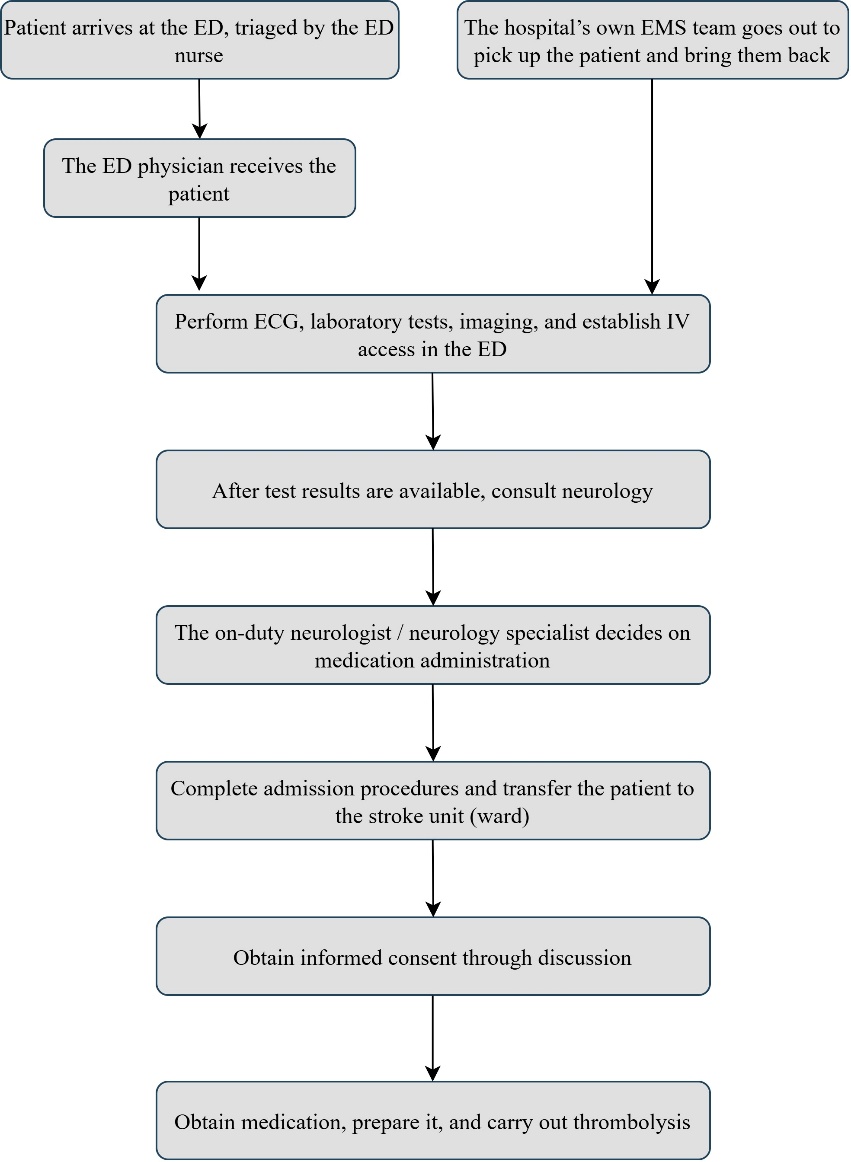


B


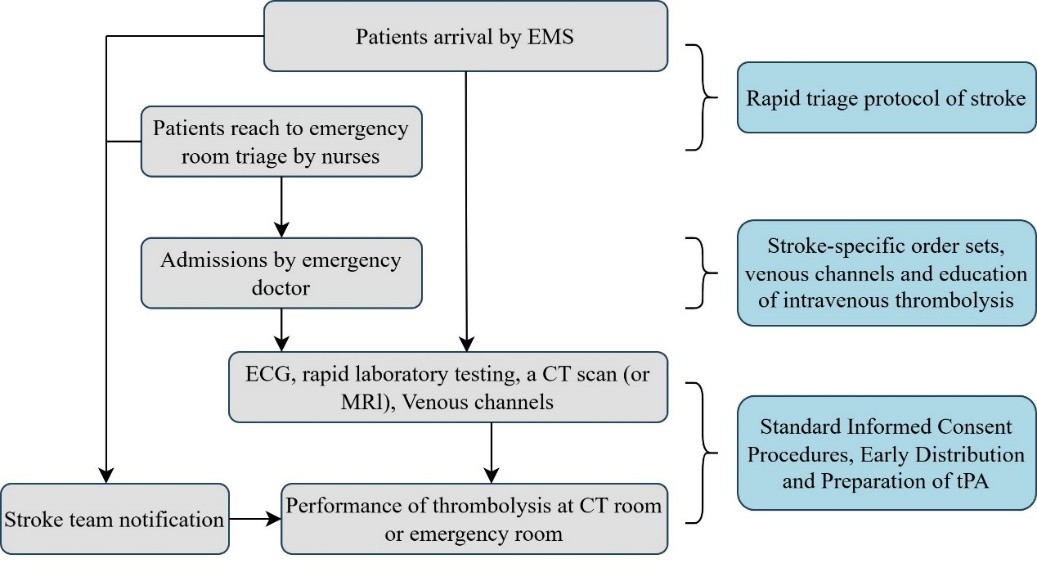


**Figure 2: Diagram of Process Reengineering for Intravenous Thrombolysis in Chinese AIS Patients. A: Current in-hospital intravenous thrombolysis process for AIS in Chinese hospitals; B: Process framework after reengineering. ED: emergency department**

3.4.2 Process Reengineering Supporting Measures

Organizational Framework Construction: Each medical center should establish a dedicated ischemic stroke thrombolysis team, including personnel from the emergency department (including pre-hospital EMS staff), neurology, neurosurgery, radiology, laboratory medicine, nurses, technicians, and other relevant healthcare professionals. The team leader for ischemic stroke thrombolysis should be a clinician experienced in cerebrovascular disease treatment. Hospital administrative intervention is encouraged to promote standardized thrombolysis construction and management, implementing continuous improvement in medical quality. Ensure that first-line neurology on-call physicians have the authority to make thrombolysis decisions or train them to acquire such authority.

Training for Stroke Team and ED Medical Staff: Conduct training every three months; provide on-demand training for new staff at any time. All training is carried out by the principal investigator of each study center or an appointed expert. Establish execution and record-keeping systems.

a) FAST stroke rapid recognition tool (Appendix 1)

b) Recommended AIS IV thrombolysis indications and contraindications (Appendix 2)

c) Application of informed consent aids for AIS IV thrombolysis

Establish a Priority Green Channel for Suspected Emergency Stroke Patients (special signage for acute stroke priority) within the Hospital: For patients who come directly to the ED, the triage nurse rapidly identifies suspected stroke patients and simultaneously activates both the ED physician and the stroke team physician. Once confirmed by the ED physician, emergency imaging is activated. For EMS personnel dispatched from the medical center, upon quickly identifying a suspected stroke, they immediately activate the stroke team and emergency imaging.

Upon arrival of an ED stroke patient, issue “Standardized Stroke Orders” (Appendix 3), and attach a special “Stroke Emergency Green Channel” marker to all requests:

a) Record vital signs (including bilateral arm blood pressure)

b) Rapid monitoring of oxygen saturation and fingerstick blood glucose

c) Establish IV access (normal saline), urgently send blood samples for CBC, coagulation screen, and emergency biochemistry (including blood glucose)

d) Issue an emergency CT scan request (Appendix 4)

Core Points of the AIS IV rt-PA Thrombolysis SOP: The stroke team should be activated as soon as possible; the stroke team should rapidly confirm the suspected stroke patient; promptly initiate informed consent discussion and medication preparation; simplify mandatory pre-thrombolysis testing; and shift the “battlefield” forward for thrombolysis.

Informed Consent SOP and Supporting Tools: A unified “Informed Consent for IV rt-PA Thrombolysis in AIS Patients” (Appendix 5); the “Health Education for IV rt-PA Thrombolysis in Acute Cerebral Infarction” public service poster (Appendix 6). From the moment the stroke team encounters the patient and identifies a possible indication for thrombolysis, begin the informed consent process, which continues from initial patient contact until imaging is completed and the informed consent form is signed.

Key Points of the Thrombolytic Drug Delivery and Preparation SOP (see Appendix 7 for details):

a) Once the stroke team member has contact with the patient and quickly determines that thrombolysis is potentially suitable, simultaneously with imaging, initiate preparation and delivery of the thrombolytic drug and necessary devices so the medication can be delivered rapidly to the ED imaging suite, while also preparing for mixing.

b) After imaging is completed and the stroke team issues the thrombolysis directive, immediately mix the medication: administer 10% of the rt-PA dose by IV push on the spot, then mix the remaining 90% in 100 mL of normal saline for IV infusion, while transferring the patient to the stroke unit.

“Real-time Feedback Form for Key Quality Control Indicators in the Ultra-early Phase of Ischemic Stroke” (Appendix 8). This form is submitted in real time to the stroke group leader at the medical center, the medical affairs department, and heads of relevant specialties, implementing real-time feedback and monitoring:

The first part includes “Time from patient arrival at ED to contact with a stroke team physician” (target <10 minutes), “Time from patient arrival at ED to start of CT/MR scanning” (target <25 minutes), “Time from patient arrival at ED to obtaining CT/MR results” (target <45 minutes), and “Time from patient arrival at ED to thrombolysis” (target <60 minutes).

The second part includes an explanation of reasons for non-thrombolysis.

Equip an AIS Emergency Toolkit: Place the FAST tool, recommended AIS IV thrombolysis indications and contraindications, posters for informed consent of IV rt-PA thrombolysis, “Informed Consent for IV rt-PA Thrombolysis in AIS Patients,” the NHSS assessment manual, the “Real-time Feedback Form for Key Quality Control Indicators in the Ultra-early Phase of Ischemic Stroke,” the AIS IV rt-PA Thrombolysis SOP, a blood pressure monitor, etc., all into one AIS toolkit. This toolkit should be stored in the ED for daily use, and the stroke team members should carry it when accompanying the patient to emergency imaging.

Each center’s principal investigator should summarize and comment on the execution of every enrolled patient’s process, identify shortcomings, and promptly provide feedback to relevant in-hospital departments and the medical affairs office. The Study Executive Committee will analyze and summarize data from all centers every three months. A phased summary will be conducted according to the characteristics of each region and hospital, and targeted improvement recommendations will be proposed to each center.

3.5 Study Phases

The study is divided into four phases of three months each, spanning a total of 12 months. The first three months serve as the preparation phase for process reengineering (baseline period), during which only key thrombolysis performance indicators are recorded. Beginning in Phase II, the newly reengineered thrombolysis process will be implemented, and a review will be conducted every three months to summarize, refine, and enhance the process (see Table 3).

| Phase I | Phase II | Phase III | Phase IV |
| --- | --- | --- | --- |
| Baseline | Post-Reengineering Intervention and Continuous Quality Improvement Stage | | |
| July–Sept 2014 | Oct–Dec 2014 | Jan–Mar 2015 | Apr–June 2015 |
| Process reengineering preparation phase | Post-Reengineering Intervention and Continuous Quality Improvement Stage | | |
| 20%-50% | >15% | >30% | >40% |
| 50% | >57% | >65% | >70% |
| 40% | >46% | >52% | >56% |
| 30% | >34% | >39% | >42% |
| <=20% | >20%-23% | >20%-26% | >20%-28% |

4. Patients Enrolled in the Study

4.1 Sample Size

Each participating center is expected to enroll an average of 10 patients per month over a 12-month period, for a total of 2,000 patients.

4.2 Inclusion criteria

1. Patients diagnosed with AIS presenting within 3.5 hours of symptom onset.

2. Written informed consent obtained from the patient or a legally authorized representative.

4.3 Exclusion criteria

1. Patients with complete resolution of neurological deficits, consistent with transient ischemic attack.

2. Patients with radiologically confirmed silent cerebral infarctions lacking clinical manifestations.

3. Patients with contraindications to intravenous thrombolytic therapy.

4.4 Treatment Plan for Patients Enrolled in This Study

This study is an interventional trial focused on the thrombolysis management process at participating centers and does not involve any additional or specific treatment recommendations. All appropriate acute-phase interventions and secondary prevention measures are provided by the attending physician in accordance with the most up-to-date domestic and international guidelines.

5. Study Endpoints

5.1 Primary outcome:

1. The median DNT before and after the intervention, as well as changes in median DNT across the four study periods

2. The proportion of patients achieving DNT <60min during the pre- and post-intervention periods

3. The proportion of intravenous thrombolysis in the pre- and post-intervention periods.

5.2 Secondary outcome:

1. Door-to-glycemic results time.

2. Door-to-biochemical results time.

3. Door-to-stroke team arrival time, representing the time spent on rapid triage and stroke team notification

4. Informed consent-to-needle time

5. Door-to-CT time

6. CT-to-needle time

5.3 Efficacy and safety outcome

**Primary Efficacy Endpoint:**

90-day mortality plus dependency (mRS 3–6) in patients receiving IV rt-PA

**Primary Safety Endpoint:**

Proportion of sICH (symptomatic intracranial hemorrhage) in patients receiving IV rt-PA, defined by NINDS criteria

**Secondary Efficacy Endpoint:**

90-day favorable outcome (mRS 0–1) in patients receiving IV rt-PA

**Secondary Safety Endpoints:**

- Proportion of sICH defined by SITS-MOST criteria in patients receiving IV rt-PA
- 90-day mortality in patients receiving IV rt-PA
- 7-day mortality in patients receiving IV rt-PA

**Additional Efficacy and Safety Endpoints:**

- Intravenous urokinase thrombolysis: efficacy and safety referenced to the above rt-PA outcomes
- Non-randomized cohort comparison between IV urokinase and IV rt-PA
- Safety and efficacy of thrombolysis in patients with NIHSS <3 or <5 (mild stroke) or those with a ≥4-point NIHSS reduction prior to thrombolysis
- Comparison of efficacy and safety between thrombolysis within 3 hours of onset vs. 3–4.5 hours
- Relationship between hemorrhagic transformation and outcomes in thrombolysis-treated patients

5.4 Prespecified Additional Endpoints and Analyses

Indirect comparative analyses with the baseline data from Phase I of the 12th Five-Year Jinqiao Project, the Phase II study data, and comparisons with the CNSR national stroke database during the 11th Five-Year Plan as well as other international stroke databases

6. Study Withdrawal

6.1 Withdrawal of Study Centers

Participating centers may withdraw from the study at any time.

6.2 Handling of Patient Withdrawal from the Registry

Each patient may terminate participation in the study at any time without providing any reason. Whenever possible, patients should discuss their decision with the investigators. The reason and time of withdrawal should be documented in the case report form (CRF). Reasons for withdrawal can be categorized as follows:
− Withdrawal without disclosing a reason (it is recommended that patients discuss their thoughts with their physician and provide a reason; if a reason is provided, it should be categorized according to the following classifications)
− Loss to follow-up
− Adverse events
− Other reasons (e.g., patient relocation or traveling abroad)
− Withdrawal initiated by the investigator for medical reasons.

6.3 Handling of Patients Who Refuse to Join the Study
This study is a non-interventional, observational cohort study. However, because part of the patient’s data will be used, written informed consent from the patient or their legally authorized representative is still required. Signing the informed consent for study participation should not cause any aversion to thrombolysis. Therefore, it is recommended that the discussion regarding study consent be held with patients or their families after admission and completion of the emergency process, explaining that only a portion of data will be collected, and to seek their consent for participation. If a patient indeed refuses, their data need not be reported in the CRF, but the first copy of the “Real-time Feedback Form for Key Quality Control Indicators in Ultra-early Ischemic Stroke” must still be submitted, and the patient should be recorded as an initially screened subject.

7. Study Follow-Up

Follow-up assessments in this study will be conducted at four time points: baseline, Day 7, at discharge, and at 90 days post-enrollment. The first three follow-ups will be reported by each study center using the CRF, while the 90-day follow-up will be performed by the study’s coordinating center using blinded telephone interviews. (See Table 4)

| **Study Visit** | **Visit 1**  **(Baseline)** | **Visit 2**  **(24–36h post-enrollment)** | **Visit 3**  **(Day 7)** | **Visit 4**  **(At Discharge)** | **Visit 5**  **(Day 90)** |
| --- | --- | --- | --- | --- | --- |
| Informed Consent | √ | — | — | — | — |
| Inclusion/Exclusion Criteria | √ | — | — | — | — |
| Demographics / Medical History | √ | — | — | — | — |
| CT/MRI | √ | √ | √ | Optional | — |
| NIHSS | √ | √ | √ | √ | √ |
| Choice of Early Antithrombotic Therapy | — | √ | √ | √ | — |
| Modified Rankin Scale | — | — | √ | √ | √ |
| Three Routine Tests and Glucose Tolerance | Optional | Optional | Optional | Optional | — |
| Blood Lipids, Blood Glucose | Optional | Optional | Optional | Optional | — |
| Major Concomitant Therapies | — | √ | √ | √ | — |
| Adverse Events / Serious Adverse Events | — | √ | √ | √ | √ |
| Time of Death and Cause Analysis | — | — | Optional | √ | √ |

Table 4: PROMISE-CHINA Follow-up Schedule and Main Assessments

8 Data Management and Statistical Analysis

All patient data will be entered and reported by investigators using a unified electronic case report form (eCRF) via the designated online reporting system. Each patient’s eCRF must be uploaded within three working days after discharge. In addition, a paper copy of the “Real-time Feedback Form for Key Quality Control Indicators in Ultra-early Ischemic Stroke” must be submitted as part of the supporting documentation. This paper form should be completed promptly on the day of stroke onset, capturing the relevant time parameters in real time and on-site. Each week, the Study Executive Committee will assign dedicated personnel to collect the first page of the paper form from each center.

Investigators are responsible for accurately and completely filling in the data required by the study’s data collection system. The eCRF, designed by the sponsor, is used to record all observed outcomes and other data pertinent to the clinical study. After all data have been entered, they must be maintained in a complete state to ensure correct interpretation. Once each patient’s eCRF is submitted online, the data manager of the Study Executive Committee will review each CRF and compare it with the paper records. Any missing or questionable parameters will be followed up by designated personnel who will visit the centers to inquire and verify data. Investigators are obligated to respond to and confirm any data queries from the Study Executive Committee. The final data entry and management will be handled by an independent Data Monitoring Committee. All data will be entered via specialized computer software to ensure accuracy.

After the database has been verified and confirmed to be correct, the Principal Investigator and members of the Data Monitoring Committee will lock the data; no further changes will be made once the data file is locked. When data entry is complete and locked, the Data Monitoring Committee will perform the required statistical analyses and prepare a statistical report, which will be submitted to the Principal Investigator of this study.

9. Modification of the Clinical Trial Protocol

All appendices attached or referenced herein are considered part of this clinical trial protocol.

No deviations or amendments to the clinical trial protocol are permitted without prior review and written approval/endorsement from the sponsoring unit of the study and the Institutional Review Board (IRB)/Ethics Committee, unless such changes are necessary to prevent immediate harm to the trial participants. Any agreed-upon amendments will be documented in writing, and the written amendment will be signed by both the investigator and the principal investigator (or study sponsor). A signed copy of the amendment will be filed with this clinical trial protocol.

Before implementing any changes, written approval/endorsement from the IRB/Ethics Committee is required for any modification to the protocol, unless an urgent safety issue necessitates immediate action. If the protocol is amended, the informed consent form may also be revised as needed. The investigator must receive IRB/Ethics Committee approval/endorsement of the revised informed consent form before implementing any changes.

10. Study Monitoring

The study’s sponsoring unit will engage an independent Contract Research Organization (CRO) to monitor the quality of the research. Any quality issues identified either through CRO monitoring or by the Study Executive Committee must be promptly addressed by the investigators to ensure high-quality conduct of the study.

11. Confidentiality

The intellectual property rights of this clinical trial protocol and all associated appendices are jointly owned by the study’s sponsoring unit(s). Investigators and their subordinate researchers are prohibited from disclosing any information without prior permission. All materials disclosed or provided by the study centers, as well as any data generated during the conduct of the clinical trial—including, but not limited to, the clinical trial protocol, the Investigator’s Manual, and the results obtained during the trial—must be kept confidential. The investigators and their subordinates agree to maintain confidentiality and shall not disclose any information to any third party without prior written approval from the principal investigator. However, this clinical trial protocol and other essential documents may be submitted to the Institutional Review Board, whose members are also subject to the same confidentiality obligations.

12. Ethics and Regulations

This clinical study will be conducted in full compliance with the ethical principles outlined in the Declaration of Helsinki (2000 version) and in accordance with all applicable Chinese laws and regulations. The study may only commence after obtaining approval of the protocol from the Institutional Review Board (IRB)/Ethics Committee. Prior to enrollment, investigators must explain the study procedures, potential benefits, risks, and patient rights to each patient (or their legally authorized representative), and must inform them that they have the right to withdraw from the study at any time without providing any reason. After obtaining full informed consent, the patient or their legally authorized representative must sign the Informed Consent Form, which should also be countersigned by the investigator conducting the consent process (including the date). Two copies of the Informed Consent Form will be produced, with one copy retained by the subject and the other by the investigator. The study will not commence until written approval from the IRB/Ethics Committee has been obtained.

During the clinical trial, any modifications or amendments to the protocol will be submitted to the IRB/Ethics Committee of the sponsoring institution. Any events that may affect patient safety or the continuation of the trial, particularly any changes regarding safety, must be promptly reported to the IRB/Ethics Committee. All updates to the Investigator’s Manual should also be submitted to the IRB/Ethics Committee. At the conclusion of the clinical trial, a summary of the study results shall be submitted to the IRB/Ethics Committee of the sponsoring institution.

13. Early Termination of the Study or Early Closure of Study Centers

**Early Termination of the Study:**

- The study will be terminated early if the number of patients arriving within 3.5 hours from onset reaches 4,000 cases;
- Alternatively, the study will be terminated early, considering a positive outcome, if all of the following three conditions are met:
  1. More than 1,500 cases have received intravenous rt-PA thrombolysis;
  2. The thrombolysis rate within 3.5 hours of onset exceeds 70%;
  3. The proportion of patients with a door-to-needle time (DNT) of <60 minutes exceeds 50%.

**Early Closure of Study Centers:**

1. The Safety Monitoring Committee will review the thrombolysis safety data of each center every three months:
   - If the incidence of symptomatic intracranial hemorrhage (sICH, as defined by NINDS criteria) exceeds 10%, or the in-hospital mortality rate exceeds 8%, the center will be subject to an investigation and corrective actions will be recommended;
   - If these rates exceed 15% and 10%, respectively, the center will have its patient enrollment suspended until clear reasons are identified and corrective measures are implemented; otherwise, the study center will be closed early.
2. A study center will also be closed early if, within one month after initiation, the investigator does not receive all the clinical trial materials required from the sponsor.
3. According to the monitoring by an independent CRO, if a center fails to enroll consecutive cases according to the standard and the rate of unreported cases exceeds 5% of the expected enrollments, corrective actions must be taken and efforts should be made to retrospectively report missing cases; if unreported cases exceed 10% of the expected enrollments, the study center will be immediately disqualified, and any cases already reported by that center will not be included in the thrombolysis rate statistics.
4. If serious data integrity issues are identified with an investigator, that study center’s qualification will be revoked, and all cases enrolled by that center will be excluded from the final statistical analysis.

14 Clinical Research Results, Research Intellectual Property, and Publication

Please refer to the research protocol (Appendix 8).

Appendix Directory:

**Appendix 1:** Rapid Stroke Screening Tool for Prehospital/Emergency Department Settings

**Appendix 2:** Indications and Contraindications for Intravenous rt-PA Thrombolysis in Acute Ischemic Stroke Patients

**Appendix 3:** Suggested Standardized Emergency Consultation and Medical Advice for Suspected Acute Ischemic Stroke Patients

**Appendix 4:** Key points of standardized operation of intravenous rt-PA thrombolysis in Acute Ischemic Stroke patients

**Appendix 5:** Informed Consent Form for IV rt-PA Thrombolysis in AIS Patients

**Appendix 6:** Health Education for IV rt-PA Thrombolysis in Acute Cerebral Infarction

**Appendix 7:** Standardized operation manual of rt-PA drug preparation for patients with suspected acute ischemic stroke

**Appendix 8:** Real time feedback form for key medical quality control indicators in the early stage of ischemic stroke

Reference

1. Xu AD, Wang YJ, Wang DZ. Consensus statement on the use of intravenous recombinant tissue plasminogen activator to treat acute ischemic stroke by the Chinese Stroke Therapy Expert Panel. CNS Neurosci Ther. 2013;19(8):543-8.

2. Jauch EC, Saver JL, Adams HP, Jr., Bruno A, Connors JJ, Demaerschalk BM, et al. Guidelines for the early management of patients with acute ischemic stroke: a guideline for healthcare professionals from the American Heart Association/American Stroke Association. Stroke. 2013;44(3):870-947.

3. Chimowitz MI. Endovascular treatment for acute ischemic stroke--still unproven. N Engl J Med. 2013;368(10):952-5.

4. El Khoury R, Jung R, Nanda A, Sila C, Abraham MG, Castonguay AC, et al. Overview of key factors in improving access to acute stroke care. Neurology. 2012;79(13 Suppl 1):S26-34.

5. Eissa A, Krass I, Levi C, Sturm J, Ibrahim R, Bajorek B. Understanding the reasons behind the low utilisation of thrombolysis in stroke. Australas Med J. 2013;6(3):152-67.

6. Zhang J, Huang Y. Treatment of acute ischemic stroke in China. Chin J Neurol. 2009;42:223-8.

7. Wang Y, Liao X, Zhao X, Wang DZ, Wang C, Nguyen-Huynh MN, et al. Using recombinant tissue plasminogen activator to treat acute ischemic stroke in China: analysis of the results from the Chinese National Stroke Registry (CNSR). Stroke. 2011;42(6):1658-64.

8. Fonarow GC, Reeves MJ, Smith EE, Saver JL, Zhao X, Olson DW, et al. Characteristics, performance measures, and in-hospital outcomes of the first one million stroke and transient ischemic attack admissions in get with the guidelines-stroke. Circ Cardiovasc Qual Outcomes. 2010;3(3):291-302.

9. Liao X, Wang Y, Pan Y, Wang C, Zhao X, Wang DZ, et al. Standard-dose intravenous tissue-type plasminogen activator for stroke is better than low doses. Stroke. 2014;45(8):2354-8.

10. Kwan J, Hand P, Sandercock P. A systematic review of barriers to delivery of thrombolysis for acute stroke. Age Ageing. 2004;33(2):116-21.

11. Zeng Y, He GP, Yi GH, Huang YJ, Zhang QH, He LL. Knowledge of stroke warning signs and risk factors among patients with previous stroke or TIA in China. J Clin Nurs. 2012;21(19-20):2886-95.

12. Yang J, Zheng M, Cheng S, Ou S, Zhang J, Wang N, et al. Knowledge of stroke symptoms and treatment among community residents in Western Urban China. J Stroke Cerebrovasc Dis. 2014;23(5):1216-24.

13. Bi Q, Zhang Z, Zhang WW, Li Q. [Study on prehospital time and influencing factors of stroke patients in 15 Chinese cities]. Zhonghua Liu Xing Bing Xue Za Zhi. 2006;27(11):996-9.

14. Mazighi M, Derex L, Amarenco P. Prehospital stroke care: potential, pitfalls, and future. Curr Opin Neurol. 2010;23(1):31-5.

15. Higashida R, Alberts MJ, Alexander DN, Crocco TJ, Demaerschalk BM, Derdeyn CP, et al. Interactions within stroke systems of care: a policy statement from the American Heart Association/American Stroke Association. Stroke. 2013;44(10):2961-84.

16. Chen S, Sun H, Zhao X, Fu P, Yan W, Yilong W, et al. Effects of comprehensive education protocol in decreasing pre-hospital stroke delay among Chinese urban community population. Neurol Res. 2013;35(5):522-8.

17. Fassbender K, Balucani C, Walter S, Levine SR, Haass A, Grotta J. Streamlining of prehospital stroke management: the golden hour. Lancet Neurol. 2013;12(6):585-96.

18. Engelter ST, Gostynski M, Papa S, Ajdacic-Gross V, Lyrer PA. Barriers to stroke thrombolysis in a geographically defined population. Cerebrovasc Dis. 2007;23(2-3):211-5.

19. Wang Y, Wu D, Zhao X, Ma R, Guo X, Wang C, et al. Hospital resources for urokinase/recombinant tissue-type plasminogen activator therapy for acute stroke in Beijing. Surg Neurol. 2009;72 Suppl 1:S2-7.

20. Meretoja A, Keshtkaran M, Saver JL, Tatlisumak T, Parsons MW, Kaste M, et al. Stroke thrombolysis: save a minute, save a day. Stroke. 2014;45(4):1053-8.

21. Ford AL, Williams JA, Spencer M, McCammon C, Khoury N, Sampson TR, et al. Reducing door-to-needle times using Toyota's lean manufacturing principles and value stream analysis. Stroke. 2012;43(12):3395-8.

22. Fonarow GC, Smith EE, Saver JL, Reeves MJ, Hernandez AF, Peterson ED, et al. Improving door-to-needle times in acute ischemic stroke: the design and rationale for the American Heart Association/American Stroke Association's Target: Stroke initiative. Stroke. 2011;42(10):2983-9.

23. Moreno A, Schwamm LH, Siddiqui KA, Viswanathan A, Whitney C, Rost N, et al. Frequent Hub-Spoke Contact Is Associated with Improved Spoke Hospital Performance: Results from the Massachusetts General Hospital Telestroke Network. Telemed J E Health. 2018;24(9):678-83.

24. Elkhuizen SG, Limburg M, Bakker PJ, Klazinga NS. Evidence-based re-engineering: re-engineering the evidence--a systematic review of the literature on business process redesign (BPR) in hospital care. Int J Health Care Qual Assur Inc Leadersh Health Serv. 2006;19(6-7):477-99.

25. Eitel DR, Rudkin SE, Malvehy MA, Killeen JP, Pines JM. Improving service quality by understanding emergency department flow: a White Paper and position statement prepared for the American Academy of Emergency Medicine. J Emerg Med. 2010;38(1):70-9.

26. Muntlin Athlin A, von Thiele Schwarz U, Farrohknia N. Effects of multidisciplinary teamwork on lead times and patient flow in the emergency department: a longitudinal interventional cohort study. Scand J Trauma Resusc Emerg Med. 2013;21:76.

27. Xian Y, Smith EE, Zhao X, Peterson ED, Olson DM, Hernandez AF, et al. Strategies used by hospitals to improve speed of tissue-type plasminogen activator treatment in acute ischemic stroke. Stroke. 2014;45(5):1387-95.
