# Supplementary material for: Impact of hospital process reengineering on door-to-needle time for intravenous thrombolysis in acute ischemic stroke (PROMISE-CHINA): a multicenter prospective pre-post quasi-experimental study
Source: Front Neurol. 2026 Apr 10;17:1746553. doi: 10.3389/fneur.2026.1746553 (PMC13105936; doi:10.3389/fneur.2026.1746553)
Supplement: Supplementary file 2 [file Supplementary_file_2.docx]

**Rapid Stroke Screening Tool for Prehospital/Emergency Department Settings**

1. **FAST scale**

**1.1 Face**

Facial Droop: Ask the patient to smile. Check for any asymmetry in facial movement; one side may appear noticeably weaker or droop.

**1.2 Arm**

Arm Weakness: Instruct the patient to raise both arms. Observe if one arm drifts downward or fails to lift fully, indicating possible weakness.

**1.3 Speech**

Speech Difficulties: Evaluate the patient’s speech for slurring, incorrect word use, or total loss of speech.

- 1. **Time**

Time Sensitivity: If any of these signs are present, it is imperative to call for emergency assistance immediately to initiate prompt treatment.

**It is recommended that emergency triage nurses promptly apply the FAST scale and simultaneously activate emergency physicians and the stroke team when facial droop, upper limb weakness, and speech impairments are observed.**

1. **Simplify prehospital Los Angeles Rating Scale (LAPSS)**

| **1** | Information/Medical History Provider | [ ] The patient | [ ] Family members | [ ] Others |
| --- | --- | --- | --- | --- |
| **2** | Time when the patient last appeared normal | Time: ___________ Date: ___________ | | |
| **Filter Criteria** | | **Yes** | **Unknown** | **No** |
| **3** | Age > 45 years old | [ ] | [ ] | [ ] |
| **4** | No history of seizures or epilepsy | [ ] | [ ] | [ ] |
| **5** | Symptom duration < 24 hours | [ ] | [ ] | [ ] |
| **6** | No history of bedridden or wheelchair dependency | [ ] | [ ] | [ ] |
| **7** | Blood glucose level between 3.3 - 22.2 mmol/L | [ ] | [ ] | [ ] |
| Physical examination (Evidence of obvious asymmetry) | | normal | left | right side |
| **8** | Facial paralysis (test: smile/grin) | [ ] | [ ] | [ ] |
|  | Weak or absent grip strength | [ ] | [ ] | [ ] |
|  | Upper limb strength (slow drooping or rapid fall) | [ ] | [ ] | [ ] |
|  | Based on the above three tests, the patient exhibits unilateral manifestations (no bilateral involvement) | Yes [ ] | No [ ] | |

**It is suggested that emergency doctors use the LAPSS to quickly assess suspected stroke patients. If options 3-8 are marked as "Yes" (or "Unknown"), the criteria for an acute stroke are met according to LAPSS, and the patient should be treated as an acute stroke case.**

**Note:** The scores derived from LAPSS are intended solely for research and screening of suspected acute stroke cases. They are not used as a clinical diagnostic tool. Even if a patient does not meet the FAST and LAPSS criteria, a diagnosis of stroke may still be made based on clinical evaluation.
