# Supplementary material for: Impact of hospital process reengineering on door-to-needle time for intravenous thrombolysis in acute ischemic stroke (PROMISE-CHINA): a multicenter prospective pre-post quasi-experimental study
Source: Front Neurol. 2026 Apr 10;17:1746553. doi: 10.3389/fneur.2026.1746553 (PMC13105936; doi:10.3389/fneur.2026.1746553)
Supplement: Supplementary file 3 [file Supplementary_file_3.docx]

**Indications and Contraindications for Intravenous rt-PA Thrombolysis in Acute Ischemic Stroke Patients**

**Indications:**

1. Acute ischemic stroke with assessable neurological deficits (such as NIHSS greater than 1 point);
2. Head imaging (CT/MRI) excludes hemorrhagic stroke;
3. Age ≥ 18 years;
4. Intravenous rt-PA can be administered within 4.5 hours of symptom onset;
5. Symptoms have lasted more than 30 minutes, or if less than 30 minutes, there is positive imaging evidence of acute cerebral infarction;
6. Patients or their families understand the potential risks and benefits of the treatment and have provided written informed consent.

Others: The American Guidelines for Early Intervention of Ischemic Stroke in 2013 and the Expert Consensus of Intravenous rt-PA Thrombolysis of China in 2012 clearly recommend thrombolysis in the following situations, although the recommended level and evidence level are not particularly high:

1. Patients with mild stroke (NIHSS below 5 points) or rapid improvement before thrombolysis;
2. Except that the low-density on CT clearly exceeds 1/3 of the middle cerebral artery area, other patients with early signs of massive cerebral infarction, such as high-density sign of middle cerebral artery, shallow sulcus or ventricular compression, thrombolysis is still beneficial.

**Contraindications:**

1) Any intracranial hemorrhage or definite history of hemorrhagic stroke; Or patients with active internal bleeding.

2) Severe neurological deficit, NIHSS>25 points (Acute basilar artery occlusion can be exempted from this restriction);

3) The low-density of CT or the high-signal area of MR-DWI is large (such as more than 1/3 of the middle cerebral artery area);

4) History of severe trauma, major surgery or massive bleeding within three months;

5) Using heparin within 48 hours before onset, aPTT exceeded the normal upper limit; Or take oral anticoagulant before onset, INR>1.6；;

Or take oral anticoagulant before onset, although INR<1.7 but the time window is 3-4.5h; *

6) Clinical consideration of subarachnoid hemorrhage, even if CT examination is normal;

7) blood sugar < 50mg/dl(2.7mmol/L)

8) Platelet count < 100,000/mm^3^ (100 * 10^9^/L); *

9) Other bleeding diseases or clear bleeding tendency. *

** Guidelines for early intervention of ischemic stroke in the United States in 2013: It is best to obtain these test results before thrombolysis, but unless there is clear evidence (such as the using of oral anticoagulants or clinical consideration of coagulation mechanism disorder), it is not necessary to delay thrombolysis because of waiting for the results.*

**Other relative taboos:**

1) Baseline blood pressure > 185/110mmHg or need intravenous drug treatment to maintain below this blood pressure standard;

2) Diabetic patients with blood sugar > 400mg/dl (21.6mmol/L) or with a history of stroke are not suitable for intravenous rt-PA thrombolysis within 3-4.5 hours after onset (thrombolysis is recommended within 3 hours after onset).

Disclaimer: The PROMISE-CHINA project team has carefully written the contents of this document, which is based on the latest, well-documented and accurate literature and practice sources. This document is only for the academic reference of the centers participating in the PROMISE-CHINA research, and the project team does not assume legal responsibility for the document itself and the consequences arising from the use of this document.
