# Supplementary material for: Impact of hospital process reengineering on door-to-needle time for intravenous thrombolysis in acute ischemic stroke (PROMISE-CHINA): a multicenter prospective pre-post quasi-experimental study
Source: Front Neurol. 2026 Apr 10;17:1746553. doi: 10.3389/fneur.2026.1746553 (PMC13105936; doi:10.3389/fneur.2026.1746553)
Supplement: Supplementary file 4 [file Supplementary_file_4.docx]

**Suggested Standardized Emergency Consultation and Medical Advice for Suspected Acute Ischemic Stroke Patients**

**Environmental requirements: Public service advertisements, such as "Early Symptoms of Suspected Stroke" and "Health Education on Intravenous rt-PA Thrombolysis for Cerebral Infarction," were displayed in the emergency department's reception hall. Additionally, "Health Education on rt-PA Thrombolysis for Cerebral Infarction" was posted in the corridor of the emergency imaging examination room.**

**All suspected stroke patients who arrive at the hospital within 3.5 hours of symptom onset should be included, following the specifications outlined below.**

1. Emergency triage nurses use FAST tools to quickly identify suspected stroke, activate emergency doctors and stroke team doctors by phone, record the clear time when patients arrive at the emergency department and the time when emergency triage nurses call the stroke team doctors. Fill in the first part of the short list of patients enrolled in PROMISE-CHINA research.
2. Emergency doctors use LAPSS tool to quickly identify suspected stroke and issue a series of standard stroke advice, in which the special signs for the green channel of stroke should be added to the application for imaging and inspection. Fill in the second part of the short list of patients enrolled in PROMISE-CHINA research.
3. Standardized medical advice and intervention parts (excluding ABC and intracranial hypertension rescue, etc.)

- Fingertip oxygen saturation; Fingertip random blood sugar; Bedside ECG and ECG monitoring (optional)
- Rapid establishment of venous access, unless hypoglycemia, glucose is not used as a carrier
- Emergency imaging application (head CT scan is preferred, head MR scan is optional)
- Blood routine, bleeding and coagulation function, basic blood biochemistry

1. Emergency doctors or nurses guide patients or their families to watch the public service advertisement of “health education of intravenous rt-PA thrombolysis in acute cerebral infarction” to inform them the possibility of thrombolysis.
2. Stroke team doctors (doctors who have the decision-making authority of thrombolysis) arrive at the patient’s bedside (the time from the patient's arrival in the emergency room to this time should be controlled within 10 minutes, and the longest time should not exceed 15 minutes).
   1. The patient should be sent to the emergency imaging examination as soon as possible after completing the preliminary examination. If the stroke team doctor has not arrived at this time, the emergency doctor should inform the stroke team doctor go to the imaging examination room directly. We should not wait for the stroke team doctor to delay the emergency imaging examination time.
   2. Stroke team members quickly confirm the suspected stroke and start the rapid preparation of drugs.
   3. Accompany the emergency imaging examination and start an informed conversation about thrombolysis that may be needed; Interpret the image inspection results immediately with the image inspectors.
   4. Make sure the diagnosis of acute ischemic stroke, make sure the indication and exclude the contraindication of thrombolysis, have a formal informed talk, and the nursing team will present or return to the emergency department. After the informed talk is signed at the first time, thrombolysis will be implemented locally or back to the emergency department.
   5. Go on to the hospitalization procedure and be admitted to the stroke unit.
   6. Start the informed conversation about whether to participate in PROMISE-CHINA research.

Disclaimer: The PROMISE-CHINA project team has carefully written the contents of this document, which is based on the latest, well-documented and accurate literature and practice sources. This document is only for the academic reference of the centers participating in the PROMISE-CHINA research, and the project team does not assume legal responsibility for the document itself and the consequences arising from the use of this document.
