# Supplementary material for: Impact of hospital process reengineering on door-to-needle time for intravenous thrombolysis in acute ischemic stroke (PROMISE-CHINA): a multicenter prospective pre-post quasi-experimental study
Source: Front Neurol. 2026 Apr 10;17:1746553. doi: 10.3389/fneur.2026.1746553 (PMC13105936; doi:10.3389/fneur.2026.1746553)
Supplement: Supplementary file 5 [file Supplementary_file_5.docx]

**Key points of standardized operation of intravenous rt-PA thrombolysis in Acute Ischemic Stroke patients**

**1. Construction of medical center system**

1.1 The stroke team was established, with senior professionals from the cerebrovascular disease clinic serving as the team leader. The team includes members from neurology, neurosurgery, the emergency department, imaging, laboratory, neurological intervention (if applicable), nursing, and technical staff. Quality control personnel from the hospital’s medical department should also be included as part of the stroke team.

1.2 The research center should implement a certification system for doctors with thrombolysis decision-making authority in ischemic stroke cases, ensuring that thrombolysis decision-makers are available 24 hours a day, 7 days a week. The time from activation to the doctor’s arrival at the bedside of patients with suspected stroke should not exceed 15 minutes.

1.3 A dedicated sign for the green channel for suspected acute stroke patients should be established. All suspected acute stroke patients who arrive at the hospital’s emergency department within 3.5 hours of symptom onset will be prioritized through the green channel. These patients will receive first-class priority throughout the hospital process, including all departments involved. This includes, but is not limited to, registration, payment, diagnostic imaging, admission, medical care, and nursing intervention. Additionally, a "special charging window for emergency stroke green channel" should be set up to streamline the process.

1.4 A training system for the rapid identification of suspected strokes should be established for emergency teams. This system should ensure that all new medical staff in the emergency department are proficient in the use of rapid stroke identification tools, such as FAST and LAPSS, as well as the overall process for managing suspected stroke cases in the emergency department.

1.5 Construction of a Stroke Healthcare Quality Monitoring and Feedback Improvement System. A quality feedback system for the reception process of suspected emergency stroke patients should be established at a minimum (refer to the Real-Time Feedback Form for Key Quality Control Indicators of Thrombolysis in Ischemic Stroke). Additionally, a primary-level system for periodic summary, feedback, and improvement must be implemented.

1.6 Publicity of Public Service Advertising System in Stroke Health Education. Posters on health education regarding intravenous rt-PA thrombolysis for acute cerebral infarction and early identification of suspected stroke should be displayed year-round in the emergency triage, emergency imaging, and neurology departments.

1.7 The research center should establish written indications and contraindications for intravenous rt-PA thrombolysis in acute ischemic stroke.

1.8 An "Acute Stroke First Aid Kit" should be reserved in the emergency department, including but not limited to the following materials:

a). Indications and contraindications of intravenous rt-PA thrombolysis in acute ischemic stroke

b). NIHSS manual

c). Poster of health education about intravenous rt-PA thrombolysis in acute cerebral infarction (A4 paper mode)

d). Informed consent of intravenous rt-PA thrombolysis in acute cerebral infarction

e). Rt-PA drug dosage form

f). Real-time feedback form of key quality control indicators of thrombolysis in ischemic stroke

g). investigator's brochure

h). Intravenous anti-hypertensive drugs

i). Sphygmomanometer

**2. Emergency triage**

2.1 When the patient arrives at the emergency department, the triage nurse records the time of arrival.

2.2 FATS tool was used to determine whether there was a suspected stroke and the onset time was initially determined. The onset time should be accurate to minute. If it is clear that the onset time is within 3.5 hours, immediately activate the stroke team and emergency doctors by phone, launch the stroke green channel and record the time (hour and minute) for activating the stroke team. Fill in the first part of the short list of patients enrolled in PROMISE-CHINA research.

**3. Emergency comprehensive intervention process of emergency doctors and nurses**

3.1 Emergency doctors use LAPSS tool to quickly identify suspected stroke and issue a series of standard stroke advice, in which the special signs for the green channel of stroke should be added to the application for imaging and inspection.

3.2 Emergency doctors and nurses: standardized medical advice and interventions part (excluding ABC and intracranial hypertension rescue, etc.)

- Measure or ask about the weight.
- Bilateral upper limb blood pressure and other vital signs.
- Fingertip oxygen saturation; Fingertip random blood sugar; Bedside ECG and ECG monitoring (optional).
- Rapid establishment of venous channel, unless hypoglycemia, glucose is not used as a carrier.
- Blood collection: blood routine, blood coagulation function and basic biochemistry, ensure that the samples can be sent to the emergency laboratory for inspection at the first time.
- Fasting (including drugs) until swallowing function test is passed.
- Unless there is a general situation that requires emergency decrease the blood pressure, do not start controlling the blood pressure when the blood pressure does not exceed 220/120mmHg, and do a good job in explaining to patients and their families.
- Apply for emergency imaging (head CT scan is preferred, head MR scan is optional), activate the emergency imaging team by phone.
- Quickly evaluate patients and exclude acute myocardial infarction, aortic dissection and other complications, or non-stroke causes (such as stroke simulation disease) and contraindications of rt-PA. Distinguish severe neurological dysfunction or potential contraindications of intravenous rt-PA;
- If the patient has respiratory distress or needs to exclude aortic or cardiac basic lesions, he should apply for emergency chest radiograph;
- Unless the airway distress requires emergency intubation, NIHSS should be scored by the stroke team before intubation;
- Quickly communicate with the stroke team members to review the treatment plan and potential contraindications of thrombolysis.
- Emergency physicians fill in the second part of the short list of patients enrolled in the PROMISE-CHINA research.

3.3 Emergency doctors or nurses guide patients or their families to watch the public service advertisement of “health education on intravenous rt-PA thrombolysis in acute cerebral infarction” to inform them the possibility of thrombolysis.

**4. Stroke team doctor (if it is consistent with the emergency doctor, it will be completed by the emergency doctor)**

4.1 Respond immediately after receiving the phone activation notice and hurry to the emergency department as soon as possible.

4.2 Communicate with emergency doctors as soon as possible, confirm suspected stroke, quickly and simply determine NIHSS*, clarify the onset time, and briefly review the indications or contraindications of intravenous rt-PA thrombolysis.

* Quick and simple determination NIHSS: Currently, there is no need for accurate inspection, only a simple assessment is needed to determine whether the score is over 25 points or within 3 points. During accompanying to emergency imaging, the accurate NIHSS should be gradually scored without delaying the examination.

4.3 Carry the "first aid kit for acute stroke", accompany the patient to the emergency imaging examination with the patient's caregiver, start the thrombolytic informed conversation that may be needed on the way and during the examination, so that the caregiver can read the “health education poster of intravenous rt-PA thrombolysis in acute cerebral infarction” and the “informed consent form of intravenous rt-PA thrombolysis in acute cerebral infarction” on the pre-reading surface, and answer and explain the questions of the caregiver.

4.4 Activated thrombolytic drug preparation process through phone call.

4.5 Read the scanning results in real time with the imaging diagnostic doctor immediately after starting emergency imaging scanning (emergency CT plain scanning is preferred), refer to the technical points:

a). Firstly, exclude the absolute contraindication of thrombolysis in imaging: cerebral hemorrhage or other intracranial hemorrhage; Low-density supply the middle cerebral artery over 1/3 area or large-area low-density foci in other areas appear. (If it is MR, it is suggested to use the high signal area in T2>1/3 area as an absolute taboo for reference).

b). After excluding the absolute contraindication of thrombolysis in imaging, the nurse can be informed immediately, so bleeding can be ruled out and the possibility of thrombolysis is extremely high. Officially start the drug preparation process, the thrombolytic drugs and their equipment will be sent to the proposed thrombolytic site immediately; If the caregiver agrees to pay the cost of 20mg rt-PA even if the thrombolysis is not performed in the end, notify the drug preparation team, open a bottle of 20mg rt-PA, and prepare 10% intravenous injection dosage.

c). Secondly, determine whether there are other imaging signs with high risk of massive cerebral infarction or thrombolytic bleeding: low density focus (not up to the above criteria); High density of middle cerebral artery; The sulcus becomes shallow and the ventricles are compressed. If yes, inform the caregiver that the relative poor prognosis and the risk of bleeding is relatively high after thrombolysis, but thrombolysis can still bring the overall benefit to the patient. Take this time point as the time to get the emergency image results!

d). Ask the imaging doctor to issue a written imaging diagnosis report and officially enable the written informed conversation to sign.

e). Formal written informed conversation is signed, meanwhile:

- Measure the blood pressure again. If the blood pressure is over 185/110mmHg, prepare intravenous anti-hypertensive drugs. After the informed consent is signed, the blood pressure should be reduced to below 185/110mmHg.
- Re-evaluation of NIHSS rapidly, determine whether there is rapid improvement or complete recovery before thrombolysis. If fully recover exist(all symptoms and signs all disappear), terminate the thrombolysis process! If it is not fully recovered, it is recommended to continue the thrombolysis process!
- Access the emergency inspection results: According to American guidelines, it is best to obtain these results before thrombolysis, unless there is a clear indicator, you should not wait for these results and delay thrombolysis.
- Get written informed consent of thrombolysis and start thrombolysis on the spot (imaging department or emergency department).

**5. Emergency imaging team/inspection team/other teams**

5.1 Upon receiving the "Emergency Stroke Green Channel" activation notification by phone, the emergency imaging department should prepare for "first-priority arrangements, including registration, equipment startup, imaging, and notifying the emergency imaging physician to be present.

5.2 Emergency radiologists should work with the stroke team doctors to interpret the emergency imaging results in real time, to ensure the correct interpretation at the first time and issue a written report as soon as possible.

5.3 Emergency examination should establish the system of "first priority" detection of samples of patients with green channel of emergency stroke and complete the report within 45 minutes after the patient arrives at the emergency department, and the sooner the better!

5.4 Specimen transportation teams and charging departments should implement the "first priority" system when they see the "green channel for emergency stroke" and establish a "special charging window for the green channel for strokes".

**6. Intravenous rt-AP thrombolysis**

6.1 Dosage: Refer to the SOP for drug preparation.

6.2 10% should be administered as an intravenous injection, with the remaining 90% given as a continuous infusion (or intravenous drip) using an infusion pump at a rate of one hour.

6.3 After completing rt-PA intravenous injection and 90% of the remaining amount, you can continue to stay in the emergency department or immediately transfer to the stroke unit of neurology for the following follow-up treatment.

a). Monitor vital signs every 15 minutes after the starting of thrombolysis and last for 2 hours; Monitor every 30 minutes for the following 6 hours; Monitoring every 1 hour for the following 16 hours;

b). Strictly control blood pressure for 24 hours according to the treatment plan;

c). Fasting before swallowing assessment;

d). Continuous monitoring the blood oxygen saturation, keeping SaO_2_ > 95% through nasal

catheter or mask;

e). Do not use antiplatelet drugs or anticoagulants in the first 24 hours (including heparin to prevent deep venous thrombosis);

f). During the first 24 hours, without indwelling catheter, nasal feeding tube, arterial or central venous catheter is allowed unless absolutely necessary;

6.4 Any acute deterioration of nervous system function after thrombolysis or obvious deterioration after improvement.

a). Immediately rescan the head plain CT.

b). If rt-PA infusion is incomplete dripped, it should continue to drip until the CT of the head determines that there is definite bleeding.

c). If no bleeding is found, other causes leading to deterioration should be found, including necessary emergency neurovascular imaging examination and necessary measures should be taken (including but not sure of the endovascular treatment that must be used).

6.5 Symptomatic hemorrhage confirmed by CT after thrombolysis;

a). Seeking neurosurgery consultation for appropriate intervention measures.

b). Laboratory test items: blood routine, PT, PTT, fibrinogen and D-dimer.

c). If fibrinogen is less than 100mg/dL, give 0.15 U/kg of cold coagulated plasma (calculated by the nearest integer). If bleeding still exists after 1 hour and the fibrinogen level is still less than 100mg/dL, give the same dose of cold coagulated plasma once again.

d). If platelet dysfunction is suspected, 4 units of platelets can be given.

6.6 When uncontrollable and life-threatening massive hemorrhage occurs, as a last attempt, 10g of aminocaproic acid can be dissolved in 250ml of normal saline for intravenous drip for more than 1 hour. Note that the use of aminocaproic acid may significantly increase the risk of pathological thrombosis.

6.7 Similar measures can be taken for severe systemic bleeding. Artificial compression can compress the bleeding site, appropriate physical methods can be sought to block the artery or vein for bleeding beyond the control of drugs.

6.8 If the above situation does not occur, CT should be reexamined 24 hours after intravenous thrombolysis, oral antiplatelet drugs should be given (if swallowing is difficult, nasal feeding tube should be given) after excluding intracranial hematoma formation or symptomatic intracranial hemorrhage. According to China expert consensus on thrombolysis with intravenous rt-PA for ischemic stroke:

a). Antiplatelet therapy should be given according to the original plan for asymptomatic hemorrhagic infarction patients although there is punctate or patchy bleeding shown on CT.

b). Patients with cerebral parenchymal hematoma or symptomatic intracranial hemorrhage should be suspended from antiplatelet therapy. The time of starting antiplatelet therapy is suggested to be made after the comprehensive evaluation of the risk of continuous bleeding and reinfarction by the competent doctor and the caregiver.

**After the thrombolysis is completed, the doctor of the stroke team will start the informed conversation and sign in whether to participate in the PROMISE－CHINA research to avoid the possible thrombolysis delay caused by this!**

**Disclaimer:** The PROMISE-CHINA project team has carefully written the contents of this document, which is based on the latest, well-documented and accurate literature and practice sources. This document is only for the academic reference of the centers participating in the PROMISE-CHINA research, and the project team does not assume legal responsibility for the document itself and the consequences arising from the use of this document.
