# Supplementary material for: Impact of hospital process reengineering on door-to-needle time for intravenous thrombolysis in acute ischemic stroke (PROMISE-CHINA): a multicenter prospective pre-post quasi-experimental study
Source: Front Neurol. 2026 Apr 10;17:1746553. doi: 10.3389/fneur.2026.1746553 (PMC13105936; doi:10.3389/fneur.2026.1746553)
Supplement: Supplementary file 6 [file Supplementary_file_6.docx]

**Informed consent of thrombolytic therapy with intravenous recombinant tissue plasminogen activator (rt-PA) in patients with acute ischemic stroke**

Dear patient/ patient's family members:

At present, the patient has been diagnosed as acute ischemic stroke (commonly known as cerebral infarction), and it meets the indications of intravenous thrombolysis with recombinant tissue plasminogen activator (rt-PA) recommended by domestic and foreign guidelines, and there is no corresponding contraindication. Till now, ultra-early (within 4.5h after onset) intravenous thrombolysis with rt-PA is the only treatment that can reduce disability and bring obvious functional recovery to patients with acute cerebral infarction. When compared with non thrombolysis, underwent rt-PA intravenous thrombolysis, one in every eight people will get a chance of complete recovery or basic recovery, and one in every three people will get a significant improvement (32 in every 100 patients will get a significant improvement)! At the same time, the increased bleeding or other harmful opportunities of rt-PA intravenous thrombolysis are far less than the huge benefits it brings: only one person in every 33 patients has bleeding or harmful opportunities.

Meanwhile, a large number of studies in mainland and abroad have proved that the earlier thrombolysis, the greater chance of benefit and the smaller of harmful risk. In developed countries such as Britain and the United States, the use of rt-PA intravenous thrombolysis for patients with ultra-early acute cerebral infarction does not require the signature and consent of patients or their families, doctors can directly use it for suitable patients. However, in view of China's national conditions and culture, we would like to inform the patients of the advantages and disadvantages of intravenous thrombolytic therapy as follows, and please decide to sign it as soon as possible (time is the brain! ), so as not to delay the treatment of patients:

**Possible benefits for patients (far exceeding risks):**

1. Dissolve of the thrombosis in the brain, symptoms improved;
2. Reduce the occurrence of progressive stroke (the deterioration of the disease);
3. Reduce patients' disability possibility.

**Potential risks and related supplements (the probability of occurrence is very small, far less than the benefits):**

1. Intracranial hemorrhage and hemorrhage in other parts of the body, very few of which may cause fatal hemorrhage;
2. Thrombosis can not dissolve, and the condition does not improve; Or after thrombolytic therapy, the blood vessels are occluded again, which makes the condition worse;
3. Allergic reactions, and even unexpected situations that endanger the life or disability of patients, such as cardiac arrest, shock and death;
4. Others:

**Informed consent statement**

I have read and understood the above information, and I have been given the opportunity to ask questions, and all my questions have been answered satisfactorily. As a patient☐/patient's family member☐, I agree☐/refuse☐ to treat the patient with rt-PA intravenous thrombolysis. Please tick “✔”or“✖”in the box to indicate your choice.

Signature of patient/patient's family member: ______________

Relationship between patient's family member to patient:______________

Signature of talking doctor:_______________ Signature time:________________

**Important Disclaimer:** Based on research evidence and recommended guidelines, the PROMISE-CHINA project team has rigorously written this informed consent form. It is recommended that the centers participating in this research use this informed consent form as a standardized thrombolysis informed conversation tool suitable for thrombolysis patients. The PROMISE-CHINA research is only a research aimed at accelerating the internal thrombolysis process management in hospitals. Whether a patient needs thrombolysis should be decided through sufficient communication between the supervising doctor and the patient or patient's family. This study does not change the patient's treatment choice. The PROMISE-CHINA project team does not assume legal responsibility for the document itself and the consequences arising from its use.
