# Supplementary figures and images for: Impact of hospital process reengineering on door-to-needle time for intravenous thrombolysis in acute ischemic stroke (PROMISE-CHINA): a multicenter prospective pre-post quasi-experimental study
Source: Front Neurol. 2026 Apr 10;17:1746553. doi: 10.3389/fneur.2026.1746553 (PMC13105936; doi:10.3389/fneur.2026.1746553)

**Health Education for IV rt-PA Thrombolysis in Acute Cerebral Infarction**

**
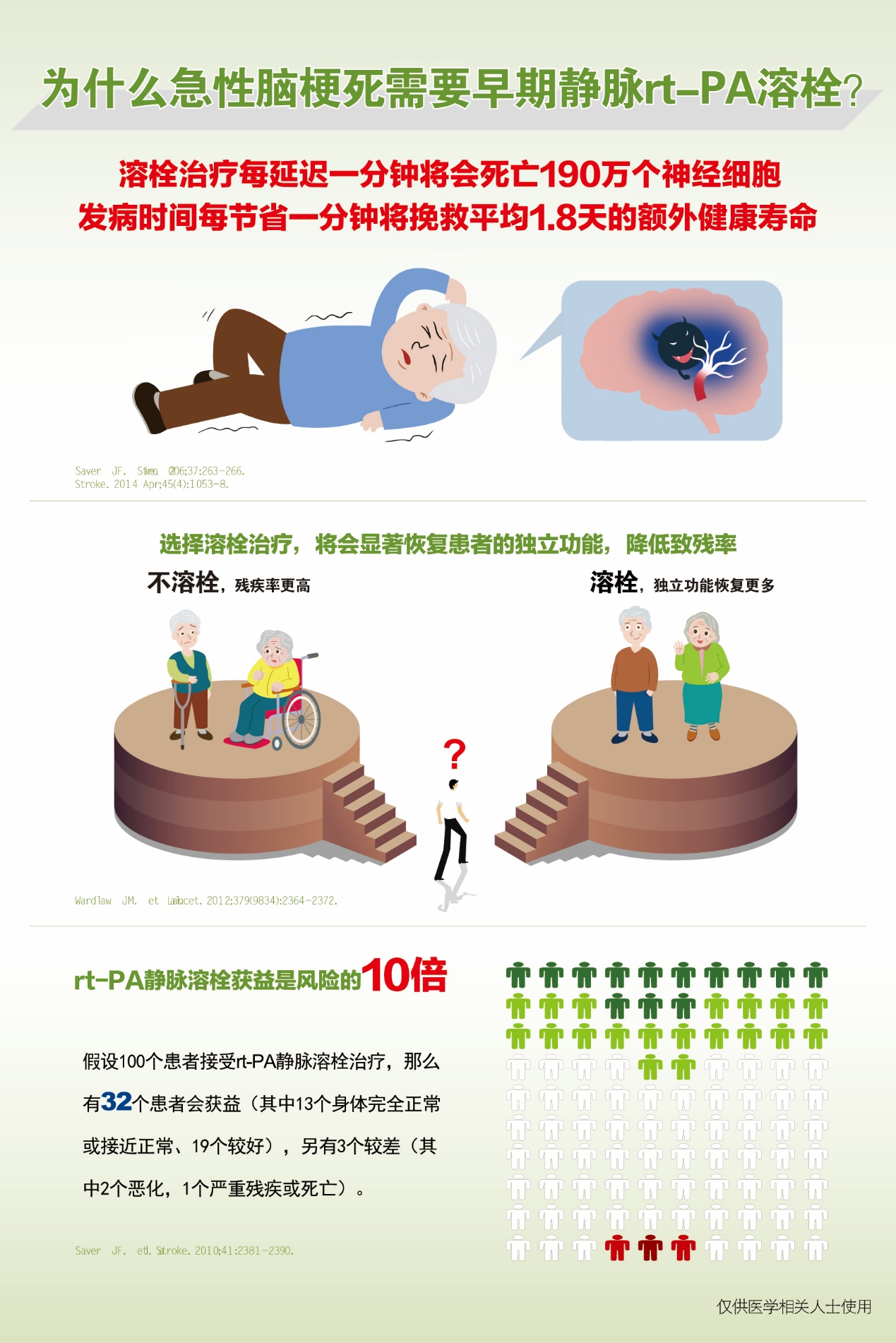

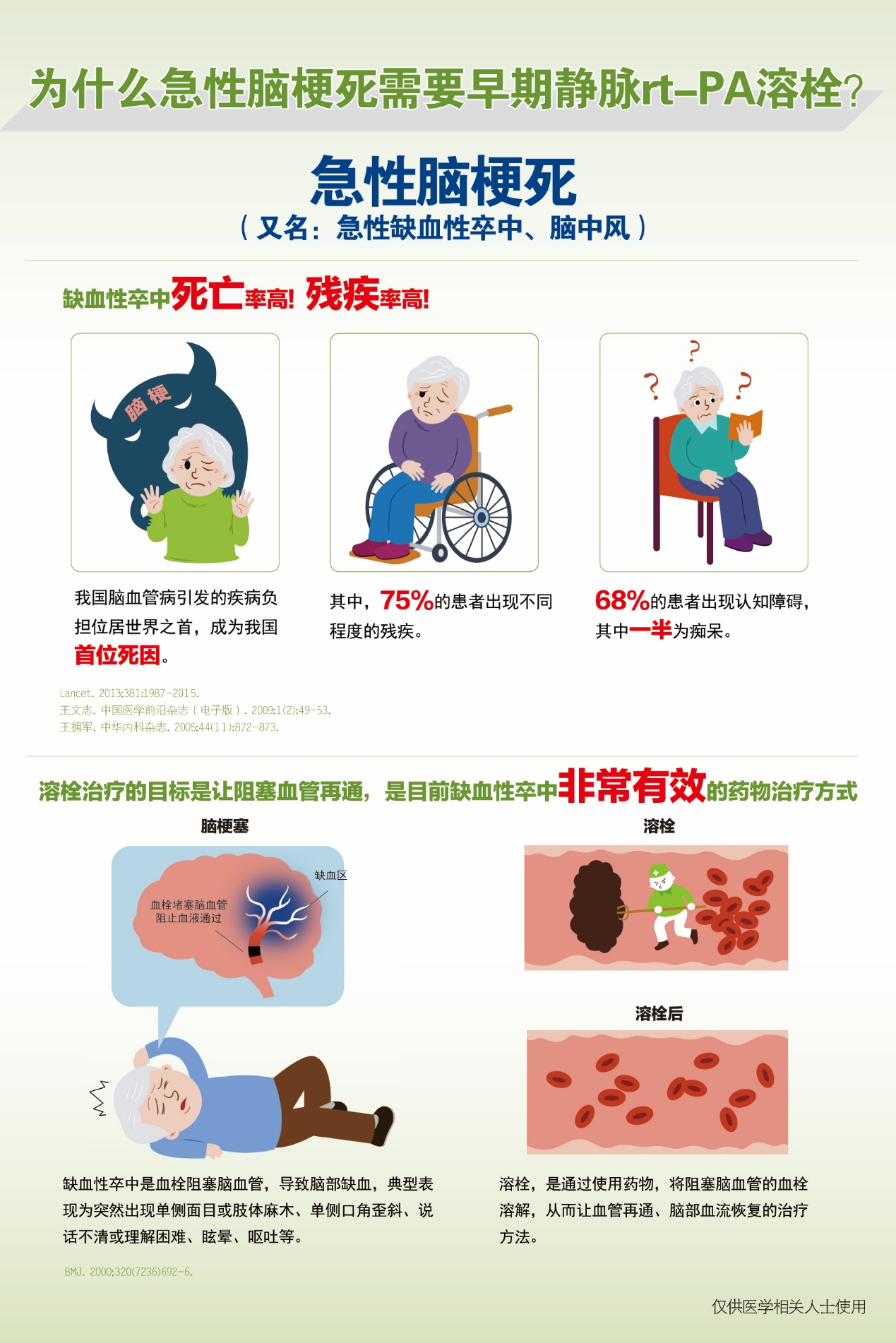
**

Supplement: Supplementary file 7 [file Supplementary_file_7.docx]
