# Supplementary material for: Impact of hospital process reengineering on door-to-needle time for intravenous thrombolysis in acute ischemic stroke (PROMISE-CHINA): a multicenter prospective pre-post quasi-experimental study
Source: Front Neurol. 2026 Apr 10;17:1746553. doi: 10.3389/fneur.2026.1746553 (PMC13105936; doi:10.3389/fneur.2026.1746553)
Supplement: Supplementary file 8 [file Supplementary_file_8.docx]

**Standardized operation manual of rt-PA drug preparation for patients with suspected acute ischemic stroke**

1. According to the characteristics of respective units, each center establish a mechanism of obtaining spare drugs at any time for 24 hours/7 days.
   1. At least two 50mg and one 20mg dosage of rt-PA are included.
   2. For unopened drugs, there should be a drug return mechanism.
   3. It is best to reserve the above spare drugs in the form of base number in the stroke unit or emergency department. A drug storage system should be established.
2. After the doctor of the stroke team arrived at the bedside of the suspected stroke patient and quickly confirmed the suspected stroke, he called the stroke nursing team to inform the patient's weight and planned rt-PA dosage (see the table below) and launched rt-PA drug preparation, including infusion sets, infusion pumps and other related equipment at the same time of emergency imaging examination. If the patient's caregiver agrees to pay the medicine fee even if he doesn't dissolve the thrombus in the end, he can use rt-PA of 20mg in advance, open a bottle of mixed medicine in advance and send it to the emergency imaging examination room.
3. After the stroke team doctor excluded bleeding through neuroimaging examination and initially determined that it was suitable for thrombolysis, he first informed the nursing team to hurry to the thrombolysis site, and formally started an informed conversation and signing with the patient/family members about thrombolysis, and then began thrombolysis after obtaining the consent.

Attached Table: List of dosage of intravenous rt-PA

| Weight (kg) | Total dose (mg) | IV dose (mg) | Weight (kg) | Total dose (mg) | IV dose (mg) |
| --- | --- | --- | --- | --- | --- |
| 40 | 36.0 | 3.6 | 72 | 64.8 | 6.5 |
| 42 | 37.8 | 3.8 | 74 | 66.6 | 6.7 |
| 44 | 39.6 | 4.0 | 76 | 68.4 | 6.8 |
| 46 | 41.4 | 4.1 | 78 | 70.2 | 7.0 |
| 48 | 43.2 | 4.3 | 80 | 72.0 | 7.2 |
| 50 | 45.0 | 4.5 | 82 | 73.8 | 7.4 |
| 52 | 46.8 | 4.7 | 84 | 75.6 | 7.6 |
| 54 | 48.6 | 4.9 | 86 | 77.4 | 7.7 |
| 56 | 50.4 | 5.0 | 88 | 79.2 | 7.9 |
| 58 | 52.2 | 5.2 | 90 | 81.0 | 8.1 |
| 60 | 54.0 | 5.4 | 92 | 82.8 | 8.3 |
| 62 | 55.8 | 5.6 | 94 | 84.6 | 8.5 |
| 64 | 57.6 | 5.8 | 96 | 86.4 | 8.6 |
| 66 | 59.4 | 5.9 | 98 | 88.2 | 8.8 |
| 68 | 61.2 | 6.1 | 100 | 90.0 | 9.0 |
| 70 | 63.0 | 6.3 | >100 | 90.0 | 9.0 |

***Note:*** *according to the weight of 0.9mg per kilogram, the maximum is no more than 90mg, 10% of the first dose is injected intravenously, and the rest is pumped intravenously for one hour. TIMS-CHINA research suggests that the above standard dose should be used for intravenous thrombolysis of rt-PA in adult acute ischemic stroke in China to obtain the maximum curative effect. The curative effect of using low dose is obviously lower than the standard dose, and the bleeding risk is the same*
