# Supplementary material for: Impact of hospital process reengineering on door-to-needle time for intravenous thrombolysis in acute ischemic stroke (PROMISE-CHINA): a multicenter prospective pre-post quasi-experimental study
Source: Front Neurol. 2026 Apr 10;17:1746553. doi: 10.3389/fneur.2026.1746553 (PMC13105936; doi:10.3389/fneur.2026.1746553)
Supplement: Supplementary file 9 [file Supplementary_file_9.docx]

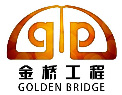
**
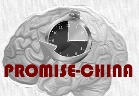
**

**Golden Bridge Special Project of the National**

**12th Five Year Plan for Science and Technology Support PROMISE-CHINA**

**Real time feedback form for key medical quality control indicators in the early stage of ischemic stroke**

Patient's name initials: ____; gender: male☐, female☐; Date of birth: ____ year ____ month ____ day

Hospitalization number: _______; Date of seeing a doctor: ____ year ____ month ____ day.

Time of onset: ____ month ____ day ____ hour ____ minute; Sleep onset: yes☐, no☐.

The onset time is unknown: Yes☐, No☐.

Call the emergency number: yes☐, no☐ ; Call time ____ hour ____ minute.

Time of arrival at the disease site: ____ hour ____ minute.

Time of patient's arrival in emergency department: ___ hour ____ minute

Time for activating the stroke team by phone: ____ hour ____ minute

Time for the stroke team to arrive at the patient's bedside: ____ hour ____ minute

Start of CT/MR examination: ____ hour ____ minute

Time of obtaining CT results: ____ hour ____ minute

Time to get blood sugar test results: ____ hour ___ minute

Time for obtaining written informed consent: ____ hour ____ minute

Start intravenous thrombolysis at ___hour ____ minute; Door to needle time：____ minute

Venous thrombolysis started site: emergency department☐imaging department☐stroke unit☐.

If intravenous rtPA thrombolysis is not performed, please fill in the specific reasons for not performing intravenous rtPA thrombolysis:

☐ Time exceeds 4.5h

☐ Time 3-4.5h, diabetes complicated with stroke in the past☐/or age > 80y☐/anticoagulation before onset☐.

☐ Implement other recanalization treatments: intravenous urokinase☐; Intra-arterial thrombolysis☐; Mechanical thrombectomy☐

☐ Other doctors think it is not suitable for thrombolysis, please elaborate:

☐ The patient or family refused, if possible, please specify the reasons for the refusal:

Name of emergency doctor _________________; Stroke team doctor's name (signature) ______________________; Application time: _______year _______ month _______ day _______ hour.

Note: The time target for the doctor of the stroke team to contact the patient when the patient arrives at the emergency department is less than 15 minutes.

The time target of patients arriving at emergency department to start CT/MR scanning is less than 25 minutes.

Patient's arrival in the emergency department-the time target of obtaining CT/MR scan results is less than 45 minutes.

The patient arrives at the emergency department-the time target of thrombolytic therapy is less than 60 minutes.

Description: This paper form is put into the portable thrombolytic first aid kit as one of the thrombolytic auxiliary tools of the portable thrombolytic first aid kit. Patients with ischemic stroke who arrive at the emergency department within 3.5h hours of onset, regardless of thrombolysis or not, must be reported continuously and fill in this form within 24 hours of the patient's visit. The first copy of this form is filed with the original case, the second copy is the research director of the research center where CRF Schedule 2 is submitted, and the third copy is submitted. The person in charge of the research center should give feedback on the information to the person in charge of the emergency department, imaging department and laboratory department in real time.

Time is the brain!

Improving the quality of emergency medical care for acute ischemic stroke, you are the power!
