# Supplementary material for: Impact of hospital process reengineering on door-to-needle time for intravenous thrombolysis in acute ischemic stroke (PROMISE-CHINA): a multicenter prospective pre-post quasi-experimental study
Source: Front Neurol. 2026 Apr 10;17:1746553. doi: 10.3389/fneur.2026.1746553 (PMC13105936; doi:10.3389/fneur.2026.1746553)
Supplement: Supplementary file 10 [file Supplementary_file_10.docx]

# **eAppendix 1: Recruitment by Site in PROMISE-CHINA**

| **NO.** | **Inclusion site** | **Number of patients recruited** |
| --- | --- | --- |
| 1 | Department of Neurology and Stroke Center, the First Affiliated Hospital, Jinan University | 33 |
| 2 | Emergency Department, Linyi People's Hospital | 172 |
| 3 | Department of Neurology, The Six Hospital of Shanxi Medical University | 82 |
| 4 | Department of Neurology, Yantai Yuhuangding Hospital | 137 |
| 5 | Department of Neurology, Zhejiang Provincial People's Hospital, Affiliated People's Hospital | 85 |
| 6 | Department of Neurology, Yantaishan Hospital | 79 |
| 7 | Department of Neurology, The First Hospital of Shanxi Medical University | 71 |
| 8 | Department of Neurology, Daping Hospital & Research Institute of Surgery, The Army Military Medical University | 82 |
| 9 | Department of Neurology, Dongyang People's Hospital, Wenzhou Medical University | 51 |
| 10 | Department of Neurology, Tieying Hospital of Fengtai District | 19 |
| 11 | Department of Neurology, First Hospital of Fangshan District | 98 |
| 12 | Department of Neurology, The First People's Hospital of Kunshan | 71 |
| 13 | Department of Neurology, Qingdao Municipal Hospital | 83 |
| 14 | Department of Neurology, The Third People's Hospital of Chengdu | 143 |
| 15 | Department of Neurology, The Second Affiliated Hospital of Soochow University | 92 |
| 16 | Department of Neurology, Qingdao Central Hospital, Qingdao University, | 55 |
| 17 | Department of Neurology, Nanjing First Hospital, Nanjing Medical University | 121 |
| 18 | Department of Neurology, Taiyuan Central Hospital of Shanxi Medical University, | 74 |
| 19 | Emergency Department, Beijing Bo Ai Hospital & China Rehabilitation Research Center, | 11 |
| 20 | Department of Neurology, Shanghai Pudong Hospital, Fudan University Pudong Medical Center | 48 |
| 21 | Department of Neurology, The Third Affiliated Hospital of Sun Yat-sen University Lingnan Hospital | 27 |
| 22 | Department of Neurology, Linyi Central Hospital | 29 |
| 23 | Department of Neurology, Zhongnan Hospital of Wuhan University | 40 |
| 24 | Department of Neurology, The People's Liberation Army No. 263 Hospital | 35 |
| 25 | Department of Neurology, Lishui Central Hospital | 63 |
| 26 | Department of Neurology, Aviation General Hospital of China Medical University & Beijing Institute of Translational Medicine, Chinese Academy of Sciences | 15 |
| 27 | Department of Neurology, The Third Affiliated Hospital of Sun Yat-sen University | 58 |
| 28 | Department of Neurology, Pingdu People's Hospital | 21 |
| 29 | Department of Neurology, Neimenggu Baogang Hospital, Inner Mongolia Medical University | 73 |
| 30 | Department of Neurology, Aerospace Central Hospital | 18 |
| 31 | Department of Neurology, Tianjin First Central Hospital of Tianjin Medical University | 10 |
| 32 | Department of Neurology, Third Hospital of Hebei Medical University | 11 |
| 33 | Department of Neurology, Shengjing Hospital of China Medical University | 8 |
| 34 | Department of Neurology, Beijing Yanhua Hospital | 25 |
| 35 | Department of Neurology, Guangzhou Red Cross Hospital, Medical College, Jinan University | 19 |
